# Neural Effects of Creative Movement, General Movement, and Sedentary Play Interventions on Interpersonal Synchrony in Children with Autism Spectrum Disorder: A Preliminary fNIRS Study †

**DOI:** 10.3390/brainsci15070683

**Published:** 2025-06-25

**Authors:** Wan-Chun Su, Daisuke Tsuzuki, Sudha Srinivasan, Anjana Bhat

**Affiliations:** 1 School of Kinesiology, Louisiana State University, Baton Rouge, LA 70802, USA; 2 Department of Information Science, Faculty of Science and Technology, Kochi University, Kochi 780-8520, Japan; 3 Physical Therapy Program, Department of Kinesiology, University of Connecticut, Storrs, CT 06269, USA; sudha.srinivasan@uconn.edu; 4 Institute for Health, Intervention, and Policy, University of Connecticut, Storrs, CT 06268, USA; 5 The Connecticut Institute for the Brain and Cognitive Sciences, University of Connecticut, Storrs, CT 06269, USA; 6 Department of Physical Therapy, University of Delaware, Newark, DE 19713, USA; abhat@udel.edu; 7 Biomechanics & Movement Science Program, University of Delaware, Newark, DE 19713, USA; 8 Department of Psychological & Brain Sciences, University of Delaware, Newark, DE 19716, USA

**Keywords:** autism spectrum disorder, functional near-infrared spectroscopy, creative movement interventions, movement intervention, neural effects, interpersonal synchrony

## Abstract

Background/Objectives: Children with Autism Spectrum Disorder (ASD) experience difficulties with interpersonal synchrony (IPS). While creative movement (CM) interventions have shown benefits for social, cognitive, and motor skills in children with ASD, the neural mechanisms underlying these improvements remain unclear. This pilot randomized control trial examined the behavioral and neural effects of CM, general movement (GM), and sedentary play (SP) interventions. Methods: Twenty-two children with ASD (Mean Age ± SE = 8.7 ± 1.9) participated. Functional Near-Infrared Spectroscopy (fNIRS) was used to measure cortical activation during a drumming synchrony task before and after 8 weeks of intervention. Results: The CM group demonstrated significant improvements in IPS and the most widespread increases in socially enhanced activation across the left middle frontal gyrus (MFG), inferior frontal gyrus (IFG), and superior temporal sulcus (STS). The GM group showed increased activation in the left IFG, while the SP group showed enhanced activation in the left STS. Children with lower baseline adaptive functioning and social responsiveness showed greater IPS improvement. Conclusions: These findings provide preliminary evidence for the efficacy of CM in improving IPS in children with ASD and support the use of fNIRS to capture neural effects following interventions.

## 1. Introduction

Autism Spectrum Disorder (ASD) is a highly prevalent neurodevelopmental disorder affecting 1 in 36 children in the US [1]. It is characterized by core challenges in social communication skills and presence of repetitive behaviors/restricted interests [2], along with co-occurring challenges during socially embedded movements, such as explicit and implicit imitation (i.e., with or without prompts to copy a demonstrator), as well as interpersonal synchrony (IPS) [3]. Difficulties in imitation and IPS in children with ASD have been linked to atypical activation patterns within the Observation Execution Matching System (OEMS), including hypoactivation in the inferior frontal gyrus (IFG) and superior temporal sulcus (STS) and hyperactivation in the inferior parietal lobe (IPL) [4,5,6]. Children with ASD might have difficulties utilizing social information during IPS tasks, as evidenced by their inability to increase OEMS activation in response to additional social cues, a response observed in typically developing (TD) peers [6]. Over the past decade, whole-body creative movement therapies (e.g., yoga, music, dance, and martial arts) have gained attention as promising interventions for addressing multisystem, social, and motor challenges of children with ASD [7]. Although creative movement therapies are known to improve social, cognitive, motor, and socially embedded motor skills (i.e., praxis and IPS) in children with ASD [7,8,9,10], the neural mechanisms underlying their positive effects remain poorly understood. Moreover, it is unclear whether and how neural activity could serve as a measure of intervention response. In this pilot randomized control trial (RCT), we investigated the neural mechanisms underlying creative movement therapies that capitalize on synchronous movements in improving the IPS performance in children with ASD. Specifically, we compared the behavioral effects of a synchrony-based creative movement (CM) intervention to those of a general movement (GM) and a standard of care sedentary play (SP) intervention and assessed cortical activation using functional Near-Infrared Spectroscopy (fNIRS) before and after the intervention in each of the three groups. It is important to note that this project is part of our ongoing effort to understand and promote IPS behaviors in children with ASD. Therefore, many of the articles cited in the following paragraphs—covering IPS behaviors, related cortical activation, and intervention effects in children with ASD—are from our research group.

### 1.1. IPS Difficulties in Children with ASD

Previous studies have reported differences in socially embedded actions (i.e., praxis and IPS) in autistic children compared to TD peers [11,12]. IPS difficulties may stem from a combination of issues related to motor incoordination, atypical social attention, and social information perception, as well as executive functioning challenges [13]. Specifically, children with ASD often exhibit fine and gross motor incoordination, poor motor planning, and difficulties with anticipatory and feedback-related motor control, which might undermine their ability to perform coordinated movements with others [14]. For instance, atypical movement characteristics, such as slower and increased variability during solo rhythmic tasks, have been correlated with IPS performance in children with ASD, highlighting the critical role of motor coordination during IPS [12]. Beyond motor coordination, children with ASD demonstrate reduced attention to social stimuli and poor biological motion perception [15,16]. These difficulties are further exacerbated during tasks requiring higher levels of social interaction. For example, children with ASD exhibit IPS performance comparable to TD peers when synchronizing movements with a computer avatar [17] but show impaired performance when synchronizing with a human [18]. Similarly, during a sway synchrony task, we found comparable performance between children with and without ASD during solo sway to a metronome but reduced IPS performance (e.g., insufficient sway amplitude and lower coherence) when swaying face-to-face with an adult tester [6]. Lastly, executive functioning, including inhibitory control and attention shifting, is also critical for synchronizing movement with others [19]. Children with ASD, who exhibit executive functioning challenges [20], may struggle to sustain goal-directed movements and spatial relations with their partner, plan and execute actions, shift attention to critical task components, and inhibit irrelevant movements to align with their partner’s actions. Collectively, these difficulties in perceptuomotor, social, and cognitive processes appear to underline the challenges children with ASD face when engaging in IPS with social partners.

### 1.2. Neural Mechanisms Underlying IPS

The OEMS, primarily comprising the IFG, STS, and IPL, plays a critical role during IPS behaviors, as it is more active when one observes, executes, and imitates/synchronizes movements with another partner [21,22,23]. The IFG and IPL interact with each other to match observed actions with one’s internal motor plans [24]. Specifically, the IFG is essential for encoding action goals, as it shows differential activation when observing actions with varying goals and is more active during the execution of goal-directed compared to non-goal-directed movements [25,26]. The IPL, on the other hand, is important for monitoring the early motor planning phases and anticipatory/predictive control of actions [27]. The STS region further supports IPS by establishing visuomotor correspondence as it becomes more active when individuals monitor and align their movements with those of a partner in an anticipatory manner [28]. Besides the OEMS, prefrontal regions (including the middle frontal gyrus (MFG)) also contribute to IPS behaviors. These regions are important for executive functioning such as motor planning, working memory, cognitive shifting, and inhibition, skills essential for successful IPS [29]. The current study examined intervention-related changes in cortical activation within the OEMS regions (i.e., IFG, STS, and IPL) and the prefrontal areas, particularly the MFG, which play an important role during executive functioning.

### 1.3. ASD-Related Differences in Cortical Activation During IPS

Research has highlighted the atypical activation of OEMS as neural correlates for imitation/IPS difficulties in children with ASD [4,30]. A comprehensive meta-analysis of functional magnetic resonance imaging (fMRI) studies revealed ASD-related hypoactivation over the IFG and STS regions, along with hyperactivation over the IPL region during imitation/IPS and its component behaviors [4]. Our prior fNIRS study similarly identified hypoactivation in the IFG and STS, along with hyperactivation in the IPL region in children with ASD during reaching and sway synchrony tasks [5,6]. Moreover, unlike their TD peers, children with ASD failed to exhibit increased cortical activation when presented with additional social visual cues during a synchronous postural sway task [6]. For instance, children with ASD did not increase cortical activation during face-to-face sway compared to solo sway which was observed in TD peers [6]. The atypical OEMS activation and absence of socially enhanced activation suggest that children with ASD may not perceive or integrate social information and utilize different neural networks during IPS tasks. In the current study, we explored intervention-related differences in socially enhanced activation (face-to-face vs. solo drumming) in the OEMS regions (i.e., IFG, STS, and IPL) and executive functioning (i.e., MFG) regions following creative movement (CM), general movement (GM), and sedentary play (SP) interventions using a pretest–post-test, RCT design.

### 1.4. Behavioral Effects of Creative Movement Interventions in Children with ASD

Studies employing creative movement interventions have reported significant improvements in various multisystem skills of children with ASD [7]. For example, our research group observed training-related improvements in joint attention, social communication/attention, gross motor performance, and imitation/praxis skills in children with ASD following yoga and music/rhythm-based interventions [8,9,10]. A comprehensive systematic review of creative movement interventions—including music, dance, yoga, theater, and martial arts—revealed small-to-large improvements in social communication after music and martial arts therapies, as well as medium-to-large improvements in motor and cognitive domains following yoga and martial arts training [7]. Despite these promising results, the aforementioned creative movement interventions vary widely in their targeted skills, intervention activities, and delivery strategies. It remains unclear which aspects of musical or rhythm-based therapies (IPS or gross-motor coordination) drive these behavioral changes. To better isolate the effects of IPS vs. gross-motor coordination practice, we included a movement comparison group that received a general movement (GM) intervention devoid of musical/rhythmic actions. To be clear, the GM intervention focused on exercise and physical endurance-based games but did not incorporate music or IPS. We aimed to compare the effects of a synchrony-based CM intervention to that of an exercise/play-based GM intervention and a standard-of-care, sedentary play (SP) intervention.

### 1.5. Knowledge Gaps in Neural Effects of Movement-Based Interventions

Compared to the extensive behavioral findings on movement interventions, evidence for the neural effects of creative movement therapies is limited. Several fMRI studies have reported training-related changes in resting-state neural activity within regions critical for social communication skills in children with ASD [31,32]. For example, Yang et al. (2021) observed increased connectivity between the left IFG and the right cerebellum after 12 weeks of mini-basketball training [31], while Sharda et al. (2018) reported reduced resting-state fMRI overconnectivity in auditory and visual regions and underconnectivity in auditory and motor regions after 8–12 weeks of music therapy in children with ASD [32]. Additionally, our systematic review of neural effects following motor interventions found that post-intervention, electroencephalogram (EEG)-based markers of cortical arousal and executive functioning improved in individuals with developmental disabilities [33]. No studies have reported neural changes in cortical activation during IPS tasks following movement interventions. The current pilot RCT investigated the behavioral and neural effects of CM, GM, and SP interventions in children with ASD during a drumming synchrony task. Furthermore, we explored correlations between IPS improvements and children’s baseline adaptive functioning, social responsiveness, and fNIRS-related cortical activation to identify subgroups that most responded to the interventions. We hypothesized that children in the CM group would exhibit the greatest intervention-related improvements in IPS performance, along with the greatest increase in socially enhanced activation compared to the GM and SP groups. Additionally, we anticipated that children with lower adaptive functioning, worse social responsiveness, and low socially enhanced cortical activation would demonstrate greater IPS improvements.

## 2. Materials and Methods

### 2.1. Participants

Twenty-four children with ASD, aged 5 to 14 years (mean age ± SE: 8.68 ± 1.65; 21 males and 3 females), participated in this study. Participants were recruited through various sources, including online announcements, phone calls, and fliers distributed to local schools, ASD services, advocacy groups, and the Simons Powering Autism Research (SPARK) participant–researcher matching service. SPARK notifies families in their database about research opportunities in the nearby area (https://www.sfari.org/resource/spark/, accessed on 23 June 2025). Prior to participation, all potential participants were interviewed to gather demographic information, including age, sex, and ethnicity, and to confirm their eligibility. Inclusion criteria required a professionally confirmed ASD diagnosis supported by a school record (e.g., documentation from a school psychologist) and/or an Individualized Education Plan (IEP) for ASD-related services. Alternatively, medical or neuropsychological records from a psychiatrist or clinical psychologist using standardized measures such as the Autism Diagnostic Observation Schedule (ADOS) and/or Autism Diagnostic Interview-Revised (ADI-R) were accepted. Additionally, the Social Communication Questionnaire (SCQ) was used to screen for ASD symptoms [34], with most children scoring above the cutoff for social communication delays (>12 points), thereby confirming the presence of ASD symptoms (Table 1). Of the 24 children initially enrolled, 2 were excluded due to a lack of post-test data (*n* = 2). Therefore, data from 22 children with ASD was included and analyzed in this study. Participants were matched based on age, sex, and level of functioning before being randomly assigned to one of the three intervention groups: CM (*n* = 8), GM (*n* = 7), and SP groups (*n* = 7). One-way ANOVAs were conducted to examine group differences in age, SCQ, VABS, and SRS scores, while Chi-square tests were used to assess group differences in sex, race, and delivery methods. No significant group differences were found for any of the variables (Table 1).

Parents of all participating children completed the Vineland Adaptive Behavioral Scales, 2nd edition (VABS-2), to provide a measure of adaptive functioning and the Social Responsiveness Scale, 2nd edition (SRS), to measure the child’s social responsiveness performance (Table 1) [35,36]. No significant differences in VABS and SRS scores were observed between the 3 groups. Written informed consent was obtained from the parents, and written/verbal assent was secured from the children before study participation. All procedures adhered to the principles of the Declaration of Helsinki and were approved by the University of Delaware Institutional Review Board (UD IRB, Study Approval #: 1539736-5). Additionally, written permission was obtained from parents and experimenters for the use of their pictures in this publication.

### 2.2. Study Procedures

**Figure 1:**
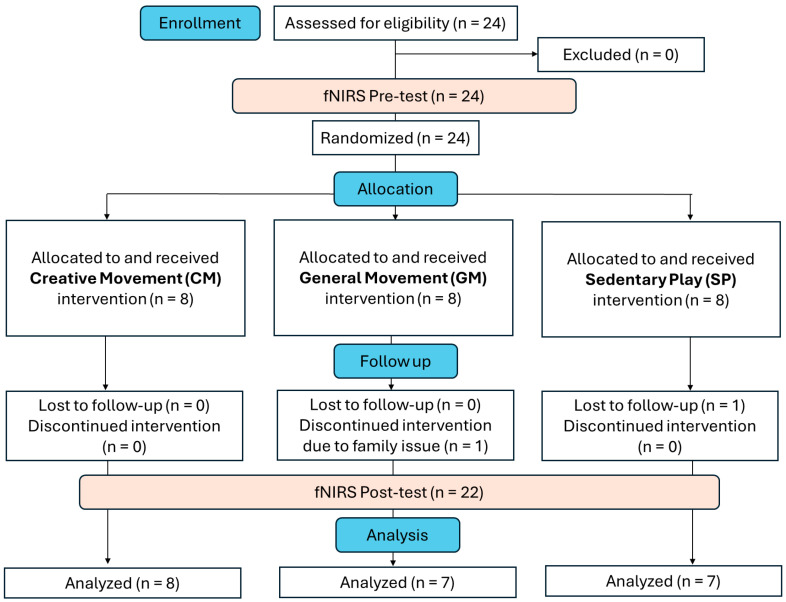
Flow diagram of the current RCT study.

The current pilot RCT spanned 10 weeks, with fNIRS pretests and post-tests conducted in the weeks immediately before and after the 8-week intervention period (Figure 1). Each child received a total of 16 training sessions (60–90 min per session; 2 sessions/week) focusing on one of the three intervention types: Creative movement (CM): Activities included music, dancing, and yoga designed to enhance social communication skills, IPS, and gross motor performance. General movement (GM): Activities included obstacle courses and strengthening/stretching exercises to improve gross motor performance. Sedentary play (SP): Activities such as reading and art–crafts targeted social communication skills and fine motor performance. The GM and SP groups served as control groups for gross motor coordination and social communication aspects within the CM intervention, respectively. Notably, the key elements of CM intervention, such as the use of musical rhythms and IPS, were excluded from the comparison interventions to isolate the unique contributions of the CM intervention. This study was conducted between 2019 and 2022, during the COVID-19 pandemic, which necessitated the use of a hybrid intervention model [37,38,39]. Participating families were given the option to choose between face-to-face (F2F) or telehealth (TH) training delivery methods. Of the participants, 12 completed the intervention F2F, while 10 completed via TH. There were no significant differences in the delivery method across intervention groups; *p* > 0.05, Table 1. Furthermore, our studies comparing the efficacy of F2F and TH delivery methods found no significant differences in intervention outcomes across all three groups [40,41,42].

### 2.3. Training Protocol

Children in all training groups participated in 16 sessions over 8 weeks (2 sessions per week), with each session lasting approximately 1 h and 15 min. The average number of sessions attended was as follows: CM: 15.1 ± 0.1; GM: 15.7 ± 0.4; SP: 15.4 ± 0.2. The average training time was as follows: CM: 78.9 ± 3.6 min; GM: 78.1 ± 7.3 min; SP: 74.2 ± 5.7 min. Each training session involved an expert trainer (i.e., a physical therapist or a developmental expert with experience working with children), a model (i.e., an undergraduate student or the parent of the child), and the participating child. All training sessions were videorecorded for later behavioral coding. For all three groups, we integrated Applied Behavior Analysis (ABA), Picture Exchange Communication System (PECS), Treatment and Education of Autistic and related Communications Handicapped Children (TEACCH), and motor learning principles to provide structure, reinforcement, and opportunities for free exploration and practice. Specifically, we offered visual, verbal, and manual prompts and positive reinforcement throughout the training period, while encouraging child choice from a set of actions or improvisation. A picture board was used to display the task schedule/order. The key components of the CM group included IPS and multi-limb, whole-body movements performed to music. During the CM intervention, the child was encouraged to move simultaneously and in synchrony with the trainer. The GM group served as a control for whole-body movement, focusing on moderate physical activities that emphasized flexibility, strength, and endurance. These activities were conducted in a turn-taking manner to ensure no obvious movement synchronization occurred. Lastly, the SP group acted as a control that mimicked the current standard of care, engaging children in desktop-based activities such as reading and fine motor tasks performed in small groups. A detailed training protocol could be found in Su et al. (2025) and Supplementary Table S1 [10]. To ensure the fidelity of training, a student coder randomly selected and coded one early (sessions 1–5) and one late session (sessions 12-16) for each child, using a fidelity checklist to assess the delivery of training activities. The training fidelity scores were consistently above 90% for all three groups (CM: 91.7 ± 0.7; GM: 91.7 ± 0.7; SP: 91.0 ± 0.9).

### 2.4. fNIRS Testing Protocol

**Figure 2:**
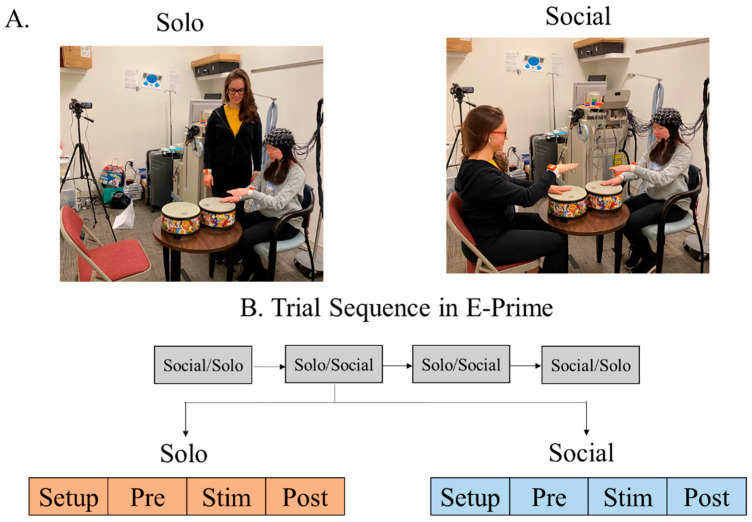
Experimental setup and trial design. (**A**) In the Solo condition, the child drummed to a metronome beat independently. In the Social condition, the child sat across from an adult tester and drummed to the metronome beat together. (**B**) A randomized block design was used in E-Prime. Each block consisted of two randomized conditions (Solo and Social), with a total of four blocks (8 trials). Each trial included a 10 s pre-stimulation period, a 25 s stimulation period, and a 25 s post-stimulation period. Written permission for publication of participant images was obtained.

Each child either sat alone at a table (Solo condition) or across from an adult tester (Social condition), with a drum/drums placed on a table (Figure 2A). An fNIRS cap, embedded with 3 × 11 probe sets, was placed on the child’s head (Figure 2A). The children completed the following conditions: (i) Solo: The child performed alternating hand movement on the drum to match various metronome patterns played in the background. The tester stood behind the child, providing no visual or social reference. (ii) Social: The child sat face-to-face with an adult tester, and together they performed alternating hand movements on the drum to match the adult’s movement patterns. To maintain the child’s engagement and prevent anticipation, the speed and rhythm of the drumming beats varied across trials. Each child completed a total of 8 trials (4 trials per condition, randomized across the session, as shown in Figure 2B). Each trial consisted of a 10 s pre-stimulation period, a 25 s stimulation period, and a 25 s post-stimulation period. During the pre- and post-stimulation periods, the child was instructed to focus on a crosshair and remain still.

### 2.5. fNIRS Data Collection

**Figure 3:**
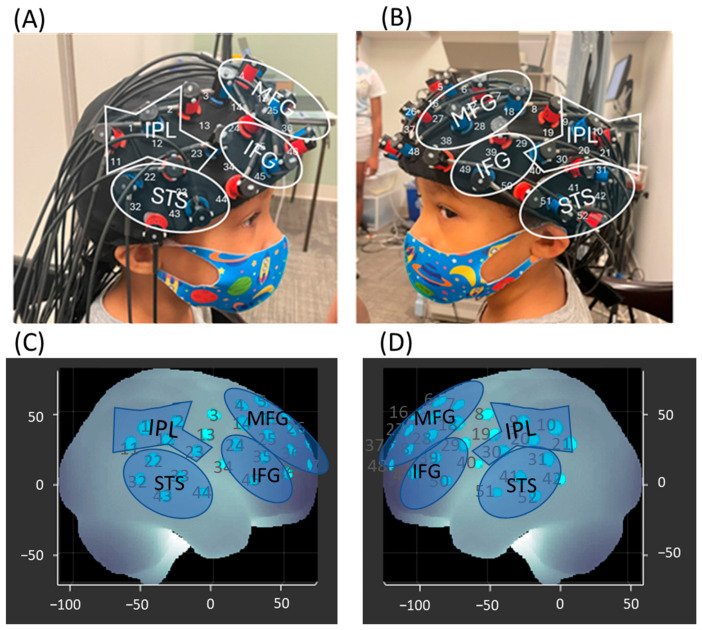
**Probe placement and channel assignment.** (**A**,**B**) Probe placement over the participant’s head. A 3 × 11 probe configuration consisting of 17 infrared emitters and 16 detectors was positioned over the bilateral frontoparietal and temporal regions. The numbered lines represent channels formed by each emitter–detector pair. (**C**,**D**) Channel assignments based on spatial registration output, indicating the corresponding regions of interest. Written permission for publication of participant images has been obtained.

Hemodynamic changes during the drumming synchrony tasks were recorded using the Hitachi ETG-4000 system (Hitachi Medical System, Tokyo, Japan; Sampling rate 10 Hz). A 3 × 11 probe set, consisting of 17 infrared emitters and 16 receivers, was positioned over the bilateral frontoparietal and temporal regions. The middle column of the probe set was aligned with the child’s nasion, while the bottom row was aligned with the child’s eyebrow (Figure 3A,B). Each adjacent pair, consisting of an emitter and a receiver, was placed 3 cm apart. The emitter emitted two wavelengths of infrared light (695 and 830 nm), which passed through the skull and created a banana-shaped arc reaching the cortical region approximately 20 mm below at the midpoint between the two probes. Thus, the midpoint of each emitter–receiver pair was treated as a channel, totaling 52 channels. Using the Modified Beer–Lambert Law, the attenuation of infrared light was used to calculate changes in concentrations of oxygenated hemoglobin (HbO_2_) and deoxygenated hemoglobin (HHb) per channel. When the cortical activation increased in a brain region, an increase in HbO_2_ concentration and a decrease in HHb concentration were expected [43]. E-Prime presentation software (version 2.0) triggered the Hitachi fNIRS system. The entire session was video recorded using a camcorder, synchronized with the Hitachi fNIRS system.

### 2.6. Spatial Registration

The ETC-4000 3D positioning unit, a reference coordinate system, was used to perform the 3D registration of the child’s cranial landmarks (nasion, inion, tragus points of the ears, and the Cz position of the International 10–20 system) and each fNIRS probe. A channel is defined as the midpoint of an emitter–receiver pair, resulting in a total of 52 channels. We applied the anchor-based spatial registration method developed by our collaborator, Dr. Tsuzuki, to transform the spatial location of each channel into the Montreal Neurological Institute (MNI) coordinate system [44]. Using the MNI coordinates, we then estimated the channel positions within a standardized 3D brain atlas based on structural information from an anatomical database of 17 adults [45]. The estimated channel locations were then labeled using the LONI Probabilistic Brain Atlas (LPBA), based on MRI scans of 40 healthy adults [46]. Each channel was considered the centroid of a sphere. A channel was included if its majority lied within a given ROI and was excluded if it did not. Additionally, a channel was excluded if its homologous counterpart belonged to a different ROI. Eventually, we assigned 40 out of the 52 channels to 4 regions of interest (ROI) on each hemisphere (Figure 3C,D; Supplementary Table S2): (i) MFG: left channels—7, 17, 18, 27, 28, 38, 48; right channels—4, 14, 15, 25, 26, 36, 47; (ii) IFG: left channels—29, 39, 49, 50; right channels—24, 35, 45, 46; (iii) STS: left channels—31, 41, 42, 51, 52; right channels—22, 32, 33, 43, 44; (iv) IPL and post-central gyrus: left channels—9, 10, 20, 21, 30; right channels—1, 2, 11, 12, 23.

### 2.7. fNIRS Data Processing

We developed custom MATLAB codes (Version: 8.1.0.604 (R2013a)) that integrated open-source software, including Hitachi POTATo (Version 3.8) and Homer-2, to process the fNIRS data [47,48]. First, we band-pass filtered the data between 0.01 and 0.5 Hz to eliminate high- and low-frequency noise related to body movements, respiration, heart rate, and other factors. We then applied the wavelet method to remove movement artifacts and used General Linear Modeling (GLM) to estimate the hemodynamic response [47,48]. To correct baseline drifts, we subtracted the linear trend between the pre-stimulation and post-stimulation baselines from values in the stimulation period. Additionally, we averaged the HbO_2_ and HHb values across channels assigned to the 8 ROIs (4 in each hemisphere). For further details, please refer to our previous publications [5,6]. To examine cortical activation in relation to changes in IPS behaviors across interventions and groups, we calculated a “socially enhanced activation” value by subtracting the average HbO_2_ level in the Solo condition from that in the Social condition. This difference was then compared across groups and time points.

### 2.8. Video Coding and Exclusion

To exclude trials with significant errors, a trained student researcher, blinded to the group assignments, screened the videos and excluded any trials in which the children did not follow the task instructions during the stimulation period. To code IPS performance, two trained student researchers independently coded 20% of the fNIRS testing videos and established intra- and inter-rater reliability (ICC > 85%). In both Solo and Social conditions, a rhythm error was coded if the child failed to follow the rhythm of the metronome beat on a second-to-second basis. In the Social condition, a synchrony error was coded if the child failed to synchronize their drumming action with the adult tester on a second-to-second basis, while a mirroring error was coded if the child did not use the mirrored hand.

### 2.9. Statistical Analysis

Normal distribution and homogeneity were assessed using Kolmogorov–Smirnov and Levene statistics, respectively. The behavioral error data did not meet the assumptions for parametric statistics; therefore, non-parametric Wilcoxon Signed-Rank Tests were used for within-group comparisons (pretest vs. post-test), and Mann–Whitney U were used for between-group comparisons (CM vs. SP, CM vs. GM, or GM vs. SP). The cortical activation data met parametric assumptions, allowing for the use of repeated-measures ANOVA. The within-group factors include time (pretest; post-test), hemisphere (left; right), and ROI (MFG, IFG, STS, and IPL), while the between-subjects factor was the group (CM, GM, and SP). To explore the potential effects of age and the intervention delivery method (i.e., F2F; TH) on intervention outcomes, we included age and delivery method as covariates. Additionally, Bayesian ANCOVA was conducted to examine the individual contribution of each factor to the significant interaction model, while Principal Component Analysis (PCA) was performed to explore the general pattern of cortical activation. Specifically, BF_10_, the Bayes Factor comparing each model to the null model, will be reported. A BF_10_ > 1 indicates greater support for the model over the null, with higher values reflecting stronger evidence. We will highlight the models with the highest BF_10_ values—specifically, those that are at least three times greater than the BF_10_ values of the competing models. To control multiple comparisons in the fNIRS data analyses, we applied the Benjamini–Hochberg False Discovery Rate (FDR) correction method [49]. Effect sizes for significant comparisons in behavioral performance and cortical activation were also reported. For the non-parametric comparisons in behavioral performance, we calculated effect sizes using the formula r = z/√N. For the normally distributed cortical activation data, effect sizes for significant post hoc comparisons were reported using Hedge’s g method [50]. Lastly, Pearson correlations were used to investigate the associations between IPS performance and cortical activation at baseline (pretest) and between improvements in IPS and adaptive functions/social communication performance (VABS; SRS), as well as fNIRS-related cortical activation at baseline. Bayesian ANCOVA and correlation analyses were conducted using JASP (version 0.19.3.0), while all other analyses were performed using SPSS (version 29.0).

## 3. Results

### 3.1. Synchrony Performance During Solo and Social Conditions

**Figure 4:**
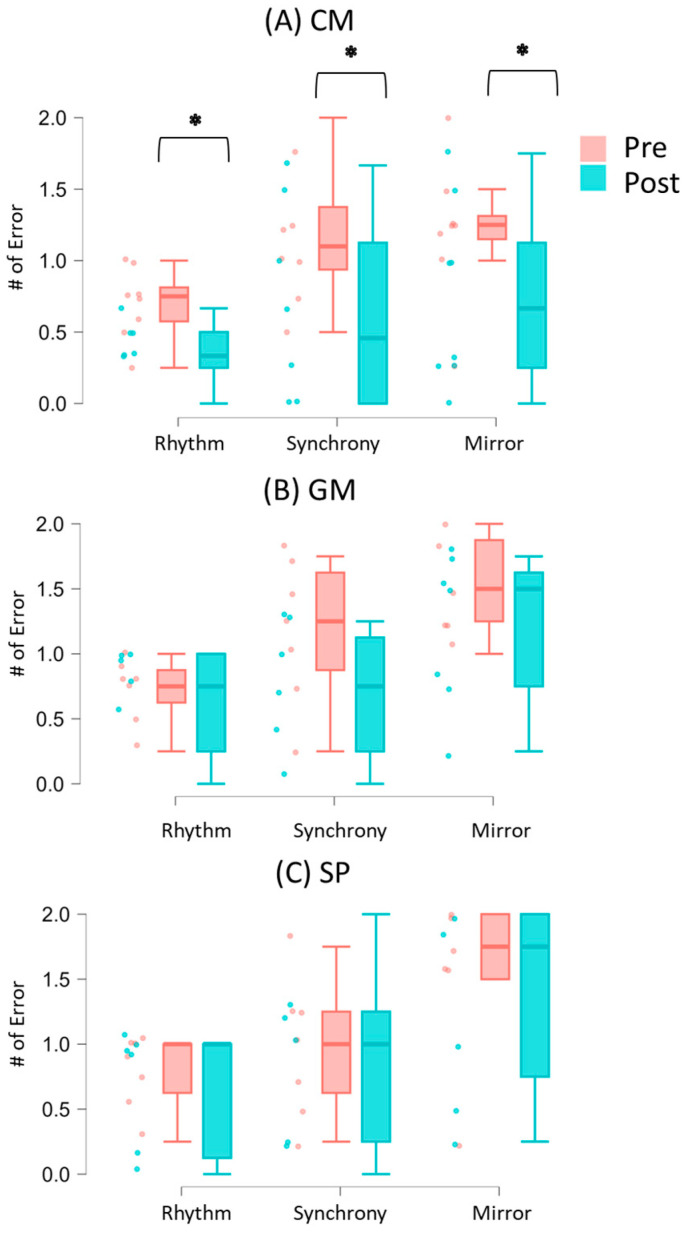
Number of rhythm, synchrony, and mirroring errors during the Social condition before (pre) and after (post) the CM (**A**), GM (**B**), and SP (**C**) interventions. Raincloud plots display individual data points with jittering, along with the median, interquartile range (IQR) boxes, and 95% confidence intervals. * indicates a significant difference in the number of errors between pre- and post-test within a group (*p* < 0.05). The CM group showed a significant decrease in rhythm error, synchrony error, and mirror error following the intervention.

The means and standard errors of synchrony performance are presented in Supplementary Table S3. In the Solo condition, none of the three groups showed significant changes in rhythm errors between the pretest and post-test (all *p*s > 0.05). In the Social condition, however, the CM group had lower rhythm, synchrony, and mirroring errors at the post-test compared to the pretest (*p*s < 0.05; r = −0.72 to −0.82), with 75% to 87.5% of the children following this trend (Figure 4A; Supplementary Table S4). Similar changes were not observed in the GM and SP groups (*p*s > 0.05; Figure 4B,C). Additionally, there were no between-group differences across errors (*p*s > 0.05; Figure 4A–C).

### 3.2. Cortical Activation During IPS

**Figure 5:**
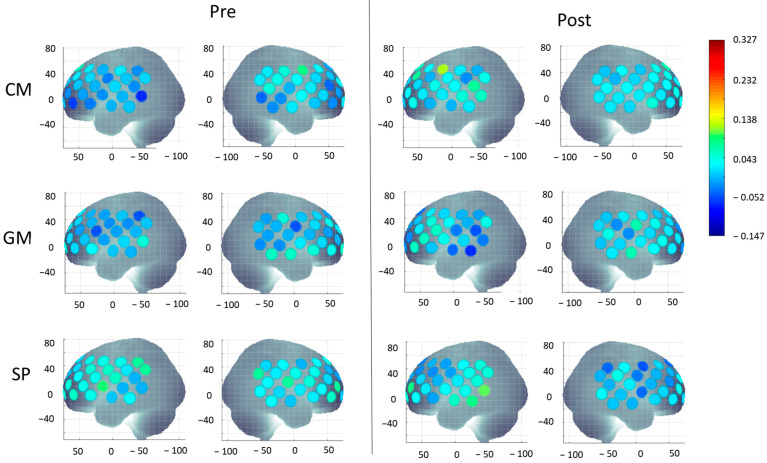
Visual representation of socially enhanced cortical activation (averaged HbO_2_ levels) before (pre) and after (post) the CM, GM, and SP interventions. Color maps display the group-averaged HbO_2_ values across individuals, with activation levels ranging from 0 (blue) to 0.327 (red), with intermediate values represented by gradient shading.

The four-way repeated measures ANOVA revealed a significant four-way interaction of Group × Time × Hemisphere × Region (*F*(4.95, 210.38) = 2.52, *p* = 0.031) for the socially enhanced activation measure. The four-way interaction did not co-vary with the covariates (i.e., intervention delivery method); therefore, post hoc analyses were completed to further explore this interaction. Bayesian ANCOVAs were conducted to further examine how Group, Time, Hemisphere, and Regions might influence the intervention outcome. The best fitting models include Time (BF_10_ = 5.477), Time + Hemisphere (BF_10_ = 3.612), and Time + Hemisphere + Time x Hemisphere (BF_10_ = 2.356). Model comparison revealed that the model including Time alone provided the strongest evidence, indicating that intervention outcomes changed over time. The addition of Hemisphere and the Time × Hemisphere interaction offered only modest improvements, suggesting that while lateralization may play a role, Time was the most robust predictor of change. Please see the full list of individual and interaction models assessed by Bayesian ANCOVA, along with their BF_10_ values, in Supplementary Table S5. Additionally, to explore the general neural activation pattern underlying the significant Group × Time × Hemisphere × Region interaction, a PCA was conducted using the cortical activation during pre- and post-intervention across all regions of interest. A Kaiser–Meyer–Olkin Measure of 0.60 and significant Bartlett’s Test of Sphericity (*p* < 0.001) support the use of PCA. A single component was extracted. Loadings for pre-intervention ROIs ranged from 0.475 to 0.740, indicating a consistent baseline activation pattern. In contrast, post-intervention ROIs exhibited weak or negative loadings (range: −0.310 to 0.244), suggesting post-intervention patterns were less aligned with the underlying component, potentially reflecting intervention-related changes in cortical activation. A detailed component matrix is reported in Supplementary Table S6. A visual representation of the averaged HbO_2_ concentration during both time points (pre- and post-intervention) and in all three groups is shown in Figure 5. The means and standard errors (SEs) of HbO_2_ concentrations are detailed in Supplementary Table S7, while the results of post hoc analyses, effect sizes, and individual trends are provided in Supplementary Table S8.

#### 3.2.1. Group Differences

**Figure 6:**
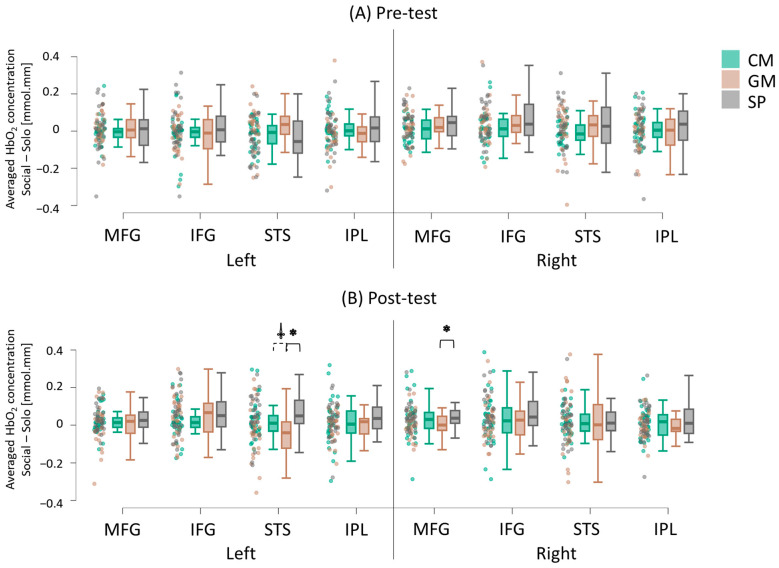
Group differences in socially enhanced cortical activation (averaged HbO_2_) before (**A**) and after (**B**) the CM, GM, and SP interventions. Raincloud plots display individual data points with jittering, along with the median, interquartile range (IQR) boxes, and 95% confidence intervals. * indicates a significant difference between groups (*p* < 0.05). ⸸ Indicates a trend toward a significant difference between groups (0.05 < *p* < 0.10). Significant group differences were observed at post-test.

At the pretest, there were no significant differences in socially enhanced cortical activation between the groups (all *p*s > 0.05; Figure 6A). However, during the post-test, children in the SP group exhibited greater socially enhanced activation in left STS and right MFG regions compared to the GM group (*p*s < 0.05; Hedge’s g = 0.08 to 0.12; Figure 6B; Supplementary Table S8). Additionally, there was a trend for greater socially enhanced activation in the left STS region for the CM group compared to the GM group during the post-test (*p* = 0.05; Hedge’s g = 0.09; Figure 6B; Supplementary Table S8).

#### 3.2.2. Intervention-Related Differences (Pretest vs. Post-Test)

**Figure 7:**
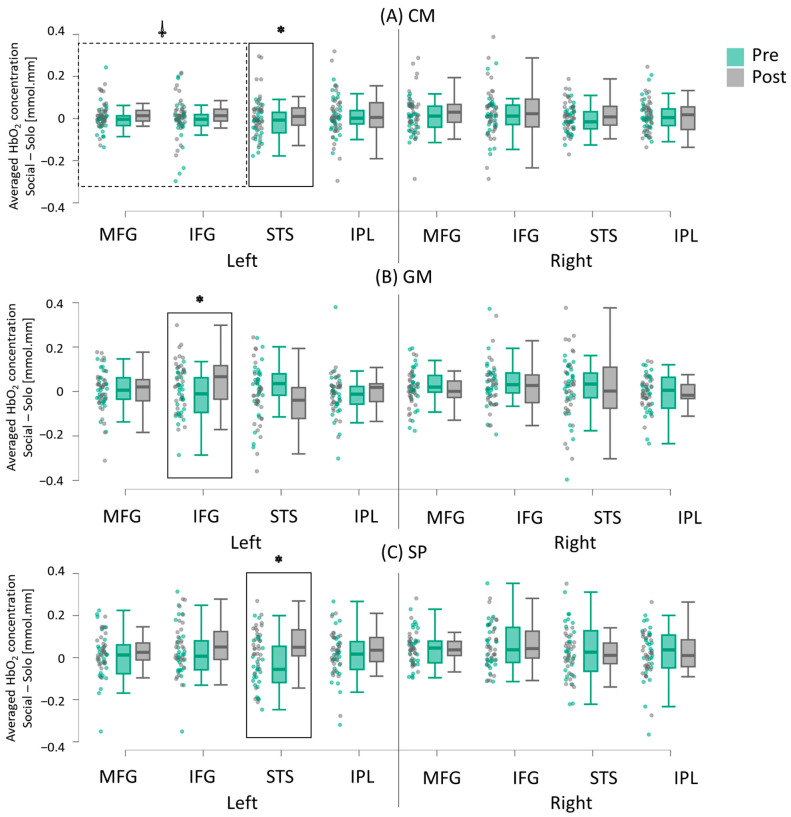
Training-related changes in socially enhanced cortical activation (average HbO_2_) in children in the CM (**A**), GM (**B**), and SP (**C**) groups. Raincloud plots display individual data points with jittering, along with the median, interquartile range (IQR) boxes, and 95% confidence intervals. Pre = pretest; Post = post-test. * indicates a significant difference between pretest and post-test (*p* < 0.05). ⸸ Indicates a trend toward a significant difference between pre-test and post-test (0.05 < *p* < 0.10). Children in the CM group exhibited the most widespread increase in socially enhanced cortical activation.

In general, all three groups demonstrated an increase in socially enhanced activation during the post-test compared to the pretest, though the specific ROIs varied across groups. In the CM group, socially enhanced activation was greater in the left STS region during the post-test compared to the pretest (*p*s < 0.01; Hedge’s g = 1.34; 87.5% of the children followed this trend; Figure 7A; Supplementary Table S8). Additionally, there was a trend for greater socially enhanced activation in the left MFG and IFG regions during the post-test compared to the pretest in the CM group (*p* = 0.094 and 0.068, respectively; Hedge’s g = 0.68 and 0.70; 100% of the children followed this trend; Figure 7A; Supplementary Table S8). In the GM group, socially enhanced activation was greater in the IFG region during the post-test compared to the pretest (*p*s < 0.05; Hedge’s g = 1.27; 71.4% of the children followed this trend; Figure 7B; Supplementary Table S8). Lastly, in the SP group, socially enhanced activation was greater in the left STS region during the post-test compared to the pretest (*p* < 0.01; Hedge’s g = 1.07; 71.4% of the children followed this trend; Figure 7C; Supplementary Table S8).

#### 3.2.3. Hemispheric Differences

**Figure 8:**
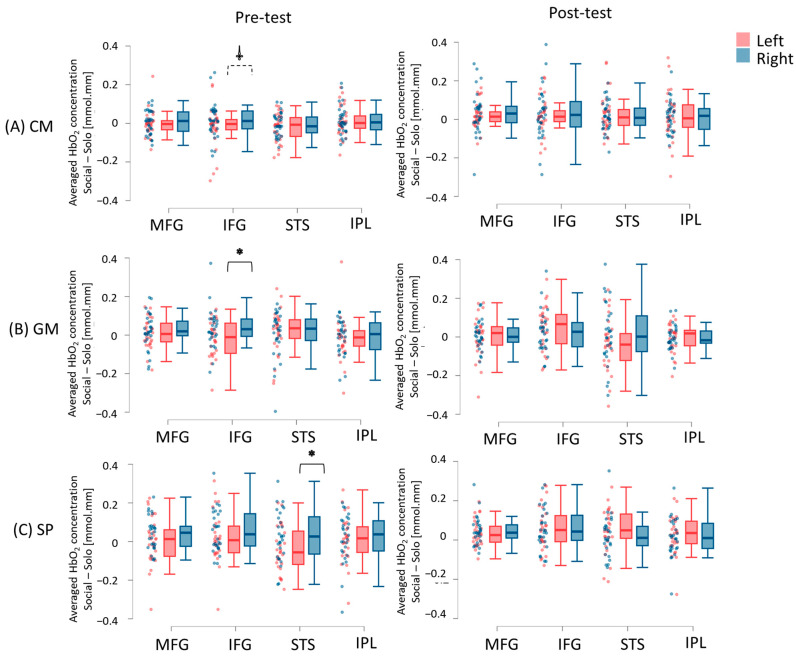
Hemispheric differences in socially enhanced cortical activation (average HbO_2_) in children in the CM (**A**), GM (**B**), and SP (**C**) groups. Raincloud plots display individual data points with jittering, along with the median, interquartile range (IQR) boxes, and 95% confidence intervals. Left = left hemisphere; Right = right hemisphere. * indicates a significant difference between left and right hemispheres (*p* < 0.05). ⸸ indicates a trend toward significant difference between left and right hemisphere (0.10 > *p* > 0.05). Greater bilateral symmetrical activation was observed at post-test compared to pretest.

All three groups showed right-lateralized socially enhanced activation (Right > Left) during the pretest, which transitioned to bilaterally symmetrical activation during the post-test. Specifically, the CM group showed a trend toward right-lateralized IFG activation during the pretest (*p* = 0.06; Hedge’s g = 0.60; 87.5% of the children followed this trend) and bilaterally symmetrical cortical activation across all ROIs during the post-test (*p*s > 0.05; Figure 8A; Supplementary Table S8). Similarly, the GM group displayed right-lateralized IFG activation (*p* < 0.05; Hedge’s g = 1.02; 71% of the children followed the trend), while the SP group showed right-lateralized STS activation during the pretest (*p* < 0.05; Hedge’s g = 1.07; 71.4% of the children followed the trend). Both groups demonstrated bilaterally symmetrical activation at the post-test (*p*s > 0.05; Figure 8B,C; Supplementary Table S8).

### 3.3. Correlation

#### 3.3.1. Associations Between IPS Performance and Cortical Activation During the Pretest

To understand whether the cortical activation reflected synchrony performance in children with ASD, we correlated the synchrony performance and cortical activation during the pretest (all participants included prior to random group assignment). In Solo condition, the children with greater rhythm errors exhibited lower IPL activation (r = −0.25; 95% CI = −0.44 to −0.04; *p* < 0.05; Table 2). In contrast, during the Social condition, children with greater rhythm errors demonstrated lower-left MFG activation (r = −0.22, 95% CI = −0.40 to −0.01; *p* < 0.05; Table 2). Only synchrony errors correlated with the activation across multiple cortical regions. Specifically, the children with more synchrony errors showed reduced activation in the left MFG, IFG, and STS regions (left MFG: r = −0.29, 95% CI = −0.47 to −0.09; left IFG: r = −0.23, 95% CI = −0.42 to −0.02; left STS: r = −0.22, 95% CI = −0.41 to −0.02; *p*s < 0.05) (Table 2).

#### 3.3.2. Associations Between Baseline Adaptive Functioning, Social Responsiveness Measures, and Improvements in IPS Performance

To explore which subgroups had greater training-related improvements in IPS performance during the drumming task, we correlated intervention-related changes in behavioral errors with baseline, adaptive functioning using VABS scores and social responsiveness using SRS scores. In the CM group, children with lower VABS scores (or lower adaptive functioning) had a greater reduction in rhythm errors (r = 0.45; 95% CI = 0.12 to 0.68; *p*s < 0.01; Table 3). In the GM group, children with higher SRS scores (or worse social responsiveness) had a greater reduction in synchrony errors (r = −0.47; 95% CI = −0.72 to −0.13; *p*s < 0.021; Table 3). In contrast, in the SP group, children with lower SRS scores (i.e., better social responsiveness) had greater reduction in Solo-rhythm errors (r = 0.42; 95% CI = 0.06 to 0.69; *p*s < 0.05, Table 3). Overall, autistic children in the CM and GM groups with lower adaptive functioning and lower social responsiveness showed greater improvements in motor/IPS performance whereas autistic children in the SP group with better social responsiveness had greater improvements in motor performance during the drumming task. The association between baseline socially enhanced activation and improvements in IPS performance is presented in Supplementary Table S9.

## 4. Discussion

Children with ASD often experience challenges in performing socially embedded movements, such as synchronizing their movements with social partners (IPS). This pilot RCT utilized a drumming synchrony task to investigate the behavioral and neural effects of CM, GM, and SP interventions in children with ASD. After 8 weeks of intervention, the CM group demonstrated significant improvements in IPS performance, reflected by fewer rhythm, synchrony, and mirroring errors. In contrast, no significant behavioral, intervention-related changes were observed in the GM and SP groups at post-test compared to pretest. With regards to cortical activation, no significant group differences were found during the pretest. However, at the post-test, the CM or SP groups exhibited greater socially enhanced activation over the left STS and right MFG regions compared to the GM group. Within-group comparisons revealed a widespread increase in socially enhanced activation in the CM group, specifically in the left MFG, IFG, and STS regions. In contrast, the GM group showed increased socially enhanced activation in the left IFG region only, and the SP group exhibited greater socially enhanced activation in the left STS region only. Hemispheric differences were also noted during the pretest, all three groups showed right-lateralized socially enhanced activation, which transitioned to bilaterally symmetrical activation at the post-test. Children with lower adaptive functioning or social responsiveness at baseline demonstrated greater improvements in synchrony performance. Overall, these findings support the preliminary efficacy of CM interventions in improving IPS performance in children with ASD and highlight the use of fNIRS for assessing intervention-related changes in cortical activation.

### 4.1. Improved IPS After Creative Movement Intervention

Consistent with our hypotheses, children in the CM group demonstrated the greatest improvements in IPS during social drumming among the three groups. Previous studies using yoga and music/rhythmic interventions have similarly reported training-related improvements in IPS skills in children with ASD [9]. Additionally, our research group conducted a comprehensive systematic review of holistic creative movement therapies (e.g., music, dance, yoga, theater, and martial arts) and highlighted multisystem benefits in children with ASD, including improvements in social communication, cognitive, and motor domains [7]. Specifically, small-to-large improvements were observed in the social communication domain following music and martial arts therapies, while medium-to-large improvements in motor and cognitive domains were found following yoga and martial arts training [7]. In the current study, we integrated dance, yoga, and music into a creative movement intervention that emphasized intense practices of IPS. The musical and dance-based activities involve observing, music-making, and joint rhythmic movements, providing rich opportunities to foster joint attention, executive functioning, perceptuomotor integration, and interpersonal connections [40]. Furthermore, the mindfulness aspects of yoga may positively influence self-regulation and executive function [41]. Taken together, the intense practice of IPS and the multi-system benefits of CM intervention likely contribute to the observed improvements in IPS performance among children with ASD.

### 4.2. Intervention-Specific Changes in Socially Enhanced Cortical Activation

Using fNIRS, this study examined changes in socially enhanced cortical activation (Activation_Social drumming_ − Activation_Solo drumming_) following CM, GM, and SP interventions. Although fNIRS does not provide structural brain information like fMRI and has lower spatial resolution compared to EEG, it offers better temporal resolution [43]. More importantly, fNIRS is more tolerant of motion artifacts. Given the movement-based nature of the drumming task used in this study, we have chosen to use fNIRS to track the intervention-related differences in cortical activation. At baseline, socially enhanced cortical activation correlated with synchrony performance variables, indicating that this activation reflected the level synchrony performance during the drumming task. Specifically, lower socially enhanced activation in the left MFG, IFG, and STS regions was significantly associated with poorer IPS performance (i.e., greater synchrony error), suggesting these areas may serve as important regions—or potential biomarkers—underlying social motor behaviors. Our preliminary findings from the repeated measures ANOVA revealed intervention-related changes in socially enhanced cortical activation across the three intervention types. Additionally, results from the Bayesian analysis and PCA suggest that Time contributed most strongly to changes in cortical activation patterns, indicating that the observed neural differences were likely driven by training-related effects rather than baseline variability. Specifically, the CM intervention led to a widespread increase in socially enhanced activation across IFG and STS, the GM intervention resulted in greater activation in IFG, and the SP intervention produced greater activation in the STS. In the following sections, we will explore the potential mechanisms driving these changes in socially enhanced activation following each intervention.

#### 4.2.1. More Widespread Increase in Socially Enhanced Activation Following CM Intervention

Among the three interventions, the CM intervention resulted in the most widespread increase in socially enhanced activation in children with ASD. Specifically, children in the CM group showed a trend toward increased activation in the left MFG and IFG, along with a significant increase in STS activation following the intervention. The observed trends (vs. significant differences) are most likely due to the small sample size per group. To address this, we conducted a post hoc power analysis using G*Power to estimate the sample sizes needed to detect significant training-related differences in left MFG and left IFG activation. The estimated sample sizes required to achieve adequate statistical power for these effects are 15 and 14 participants, respectively. The widespread increase in activation is not surprising, as CM interventions incorporate both the motor coordination components emphasized within the GM intervention and the social/synchronous actions required for IPS. For example, during dance activities, children performed whole-body movements and various locomotor skills (e.g., jumping; skipping) while following the trainer. During musical activities, children shifted attention between the instruments they played and those played by the trainers, fostering joint attention to create harmonious music together. Most importantly, the CM intervention emphasizes IPS, which involves precise synchronization and matching of movements between the child and the trainer. As previously discussed, the OEMS, including the IFG and STS regions, plays a crucial role in perceiving and monitoring others’ actions and coordinating one’s own actions accordingly [22]. Our previous fNIRS study indicated that children with ASD did not scale up IFG and STS activation when provided with social visual information during synchronized swaying, unlike their TD peers [6]. The increased socially enhanced activation over the IFG and STS regions observed in the current study likely reflects intervention-related improvements in perceiving social cues and synchronizing/matching their own actions to others.

Executive functioning—a set of cognitive skills encompassing motor planning, working memory, attention shifting, and response inhibition—is another key area targeted during the CM intervention. For example, in the musical chairs game (where children and the trainer move when the music plays and freeze when it stops), inhibitory control is challenged. Similarly, attention shifting is challenged when children adjust their movement patterns based on changes in musical rhythms, such as performing smooth movements (e.g., swaying) for quiet music and sharp movements (e.g., punching) for syncopated music. The MFG, IFG, and other prefrontal regions are important for executive functioning [29]. Therefore, the observed increase in socially enhanced activation in these areas may reflect a heightened focus on executive functioning during social drumming. In summary, the widespread increase in socially enhanced activation across the OEMS regions (i.e., inferior frontal and superior temporal) regions following CM intervention likely reflects improvements in motor coordination, joint attention, perceiving/matching of social information, and executive functioning in children with ASD.

#### 4.2.2. GM Intervention-Related Increase in Socially Enhanced Activation in IFG

Children in the GM group demonstrated greater socially enhanced activation in the IFG region following the intervention. As noted previously, IFG, along with other prefrontal cortex regions, plays a critical role in executive functioning [29,42]. The GM intervention specifically targets executive functioning. For example, children are required to plan and execute various locomotor movements (i.e., motor planning) and adapt by changing moving directions or movement types (i.e., mental flexibility) while navigating an obstacle course. More specifically, during the red-light, green-light game, children had to stop traveling/freeze and catch a ball thrown at them, further challenging their inhibitory control. In summary, the finding of increased socially enhanced activation in the IFG region might reflect improvements in executive functioning as a result of the GM intervention.

#### 4.2.3. SP Intervention-Related Increase in Socially Enhanced Activation in STS

On the other hand, children in the SP group showed greater socially enhanced activation in the STS regions following the intervention. As a key component of the OEMS, the STS region plays an important role in establishing visuomotor correspondence and is activated when individuals monitor and match their actions with those of their partners in either a predictive or reactive manner [28]. The STS is particularly sensitive to biological motion [51] and is implicated in gaze-following behavior and the facilitation of joint attention [52], both of which are foundational to social and motor learning. During the SP intervention, children were required to closely attend to the trainer’s demonstrations in order to learn and perform fine motor skills. This involved not only observing and imitating object-related hand motions but also referring to visual instructional materials that presented step-by-step guidance for completing building and art–craft activities. These tasks demanded sustained visual attention, hand–eye coordination, and action imitation—key processes linked to STS functioning. The observed increase in socially enhanced activation in the STS likely reflects the children’s improvement in interpreting and mimicking goal-directed hand actions in a socially relevant context. Together, these findings highlight the potential of structured seated play activities to support social–motor learning by strengthening neural mechanisms associated with action observation and imitation.

### 4.3. Increased Left Lateralization Post-Movement Interventions

Training-related differences in socially enhanced activation were predominantly observed in the left hemisphere. Additionally, the right-lateralized socially enhanced activation evident at baseline became more symmetrical following all three types of interventions, suggesting greater left hemisphere activation during the intervention as well as the drumming task. Previous research has indicated that children with ASD often struggle with functions primarily represented in the left hemisphere, such as social communication, language, and motor control, while showing relatively intact right hemisphere functioning [53]. Despite their focus on different skill sets, all three interventions targeted the left hemisphere functions. For example, whole-body motor control is a core component of the GM and CM interventions, while language and social communication (verbal and non-verbal) skills were integral to the delivery of CM, GM, and SP interventions. The intensive practice of these left hemisphere functions may have contributed to the more symmetric, socially enhanced activation and improvements in both social and motor performance.

### 4.4. Limitations and Future Directions

The current pilot RCT had a small sample size, which limited our ability to detect smaller training effects and to examine which subgroups—based on factors such as age, sex, and level of functioning—benefited most from the three interventions. Additionally, we did not examine how prior motor experience, intelligence level, or concurrent therapies may have influenced outcomes, nor did we include typically developing controls to further evaluate the effects of the interventions. Future studies should build on this work by including a diverse range of subgroups (e.g., younger vs. older children those requiring varying levels of support) as well as typically developing controls. Additionally, due to the COVID-19 pandemic, we adopted a pragmatic approach by offering a hybrid intervention model that allowed participating families to choose between face-to-face (F2F) or telehealth (TH) delivery methods [37,38,39]. While delivery methods did not show significant effects in our ANOVA analyses of cortical activation, and our prior studies comparing the efficacy of F2F and TH delivery methods revealed no significant differences in intervention-related outcomes [37,38,39]; future studies with larger sample sizes should further explore the potential impact of delivery methods on intervention outcomes. It is also important to note that due to the cyclic and rhythmic nature of the drumming task, it is challenging to capture a broader range of error values using our current coding scheme. Future studies should incorporate biomechanical measures to more precisely quantify synchrony behaviors. Lastly, we did not include children who dropped out from the GM (n = 1) and SP (n = 1) groups in the analyses. Future studies should consider using intention-to-treat analyses to account for dropouts to improve robustness of findings.

## 5. Conclusions

The current RCT reaffirms the preliminary efficacy of creative movement interventions in improving IPS performance in children with ASD, using behavioral and neural measures. Additionally, it highlights the potential of fNIRS as a valuable tool for assessing intervention-related changes in cortical activation outcomes. Clinicians are encouraged to integrate whole-body movements, including music and rhythmic activities, into routine interventions for children with ASD. They may also consider using fNIRS-related functional activation outcomes to monitor intervention progress following movement interventions.

## Figures and Tables

**Table 1:** Demographic information and the results of social communication questionnaires in ASD and TD groups. Means and standard errors (SE) provided.

Characteristics	CM Group (*n* = 8) Mean ± SE	GM Group (*n* = 7) Mean ± SE	SP Group (*n* = 7) Mean ± SE	Between Group Comparison (*p*-Value)
Age	9.50 ± 0.96	8.42 ± 0.95	8.56 ± 0.94	*F*(2, 21) = 0.62, *p* = 0.55
Sex	6 M, 2 F	7 M	6 M, 1 F	X^2^ (2, N = 22) = 1.99, *p* = 0.37
Race/Ethnicity	6 C, 2 AAC	3C, 3A, 1AA	5 C, 2 AA	X^2^ (8, N = 22) = 13.92, *p* = 0.08
Delivery Method	5 F2F, 3 TH	3 F2F, 4 TH	4 F2F, 3 TH	X^2^ (2, N = 22) = 0.61, *p =* 0.74
SCQ	17.25 ± 2.18	12.57 ± 1.66	12.29 ± 1.19	*F*(2, 21) = 2.56, *p* = 0.10
VABS-II (SS)	70.25 ± 3.59	77.43 ± 3.45	75.89 ± 4.04	*F*(2, 21) = 2.25, *p* = 0.13
VABS-II Communication	73.50 ± 3.70	81.71 ± 4.77	82.00 ± 6.00	*F*(2, 21) = 2.20, *p* = 0.14
VABS-II Daily living	71.75 ± 4.03 ^a^	80.57 ± 4.44	81.11 ± 3.73	*F*(2, 21) = 2.68, *p* = 0.09
VABS-II Socialization	70.25 ± 3.59	80.14 ± 2.87	69.11 ± 6.55	*F*(2, 21) = 2.53, *p* = 0.11
SRS (T scores)	77.88 ± 4.25	68.14 ± 2.84	73.11 ± 2.05	*F*(2, 21) = 2.07, *p* = 0.15
SCI (T scores)	74.50 ± 4.02	67.14 ± 2.52	70.78 ± 2.98	*F*(2, 21) = 1.40, *p* = 0.27
RRB (T scores)	79.63 ± 3.17	68.86 ± 4.79	72.00 ± 3.54	*F*(2, 21) = 1.94, *p* = 0.171

VABS-II = Vineland Adaptive Behavior Scale—2nd Edition; SS = Standard Score; SRS = Social Responsiveness Scale; SCI = Social Communication and Interaction; RRB = Restricted Interests and Repetitive Behavior; M = Male, F = Female; C = Caucasian, A = Asian, AA = African American, AAC = African American-Caucasian; ^a^ indicates significant differences between children in the CM compared to the SP group (*p* < 0.05).

**Table 2:** Correlations between IPS performance and cortical activation during the pretest.

r-Values	Solo	Social		
	Rhythm	Rhythm	Synchrony	Mirror
Left hemisphere				
MFG	−0.05 (−0.26 to 0.16)	−0.22 (−0.40 to −0.01) *	−0.29 (−0.47 to −0.09) *	−0.03 (−0.23 to 0.18)
IFG	−0.15 (−0.35 to 0.07)	0.01 (−0.19 to 0.22)	−0.23 (−0.42 to −0.02) *	0.09 (−0.12 to 0.29)
STS	−0.09 (−0.29 to 0.13)	−0.18 (−0.37 to 0.03)	−0.22 (−0.41 to −0.02) *	−0.03 (−0.24 to 0.18)
IPL	−0.25 (−0.44 to −0.04) *	−0.13 (−0.33 to 0.08)	−0.20 (−0.39 to 0.01)	−0.04 (−0.25 to 0.17)
Right hemisphere				
MFG	0.02 (−0.19 to 0.23)	−0.11 (−0.31 to 0.10)	−0.13 (−0.32 to 0.08)	0.16 (−0.05 to 0.36)
IFG	−0.07 (−0.27 to 0.15)	−0.02 (−0.22 to 0.19)	−0.02 (−0.23 to 0.19)	0.06 (−0.15 to 0.26)
STS	−0.10 (−0.30 to 0.12)	−0.01 (−0.22 to 0.20)	−0.10 (−0.30 to 0.11)	0.15 (−0.06 to 0.35)
IPL	−0.13 (−0.33 to 0.08)	−0.05 (−0.26 to 0.16)	0.04 (−0.17 to 0.24)	0.14 (−0.07 to 0.33)

R values (and their 95% confidence intervals) are presented. * indicates a *p*-value < 0.05.

**Table 3:** Correlations between baseline adaptive functioning, social responsiveness measures, and improvements in IPS performance.

r-Values	Solo	Social		
	Δ Rhythm	Δ Rhythm	Δ Synchrony	Δ Mirror
CM Group				
VABS (SS)	0.45 (0.12 to 0.68) *	−0.04 (−0.38 to 0.31)	−0.13 (−0.46 to 0.22)	−0.04 (−0.38 to 0.31)
SRS (T score)	−0.33 (−0.61 to 0.01)	0.10 (−0.25 to 0.43)	0.18 (−0.18 to 0.49)	−0.17 (−0.49 to 0.18)
GM Group				
VABS (SS)	−0.10 (−0.45 to 0.27)	−0.04 (−0.40 to 0.33)	0.03 (−0.34 to 0.39)	−0.24 (−0.56 to 0.14)
SRS (T score)	−0.07 (−043 to 0.30)	0.08 (−0.30 to 0.43)	−0.47 (−0.72 to −0.13) *	0.03 (−0.34 to 0.39)
SP Group				
VABS (SS)	−0.05 (−0.41 to 0.33)	−0.23 (−0.55 to 0.16)	−0.31 (−0.61 to 0.08)	−0.12 (−0.47 to 0.27)
SRS (T score)	0.42 (0.06 to 0.69) *	−0.13 (−0.48 to 0.26)	0.14 (−0.25 to 0.49)	0.24 (−0.15 to 0.56)

R values (and their 95% confidence intervals) are presented. Δ indicates changes in behavioral performance after intervention. * indicates a *p*-value < 0.05.

## Data Availability

The data presented in this study are available on request from the corresponding author. The data are not publicly available due to restrictions associated with participants’ privacy.

## Supplementary Materials

The following supporting information can be downloaded at https://www.mdpi.com/article/10.3390/brainsci15070683/s1: Table S1: Conditions, examples of training activities, and skills promoted during exemplar CM, GM, and SP intervention sessions; Table S2: Assignment of channels to regions based on spatial registration; Table S3: Means and standard errors of synchrony performance in children in CM, GM, and SP groups; Table S4: Summary of *t*-test analyses, effect sizes, and the individual trends for the synchrony performance; Table S5: Summary of the Bayesian ANCOVA analyses; Table S6: Component matrix for Principal Component Analyses; Table S7: Means and standard errors of Social–Solo HbO_2_ concentration in children in CM, GM, and SP groups; Table S8: Post hoc analyses of Social–Solo cortical activation for the Group × Time × Hemisphere × Region 4-way interaction; Table S9: Correlation between baseline socially enhanced activation and improvements in IPS performance.
