# Supplementary material for: Neural Effects of Creative Movement, General Movement, and Sedentary Play Interventions on Interpersonal Synchrony in Children with Autism Spectrum Disorder: A Preliminary fNIRS Study
Source: Brain Sci. 2025 Jun 25;15(7):683. doi: 10.3390/brainsci15070683 (PMC12293324; doi:10.3390/brainsci15070683)
Supplement: Supplementary file 1 [file brainsci-15-00683-s001.zip › brainsci-3679216-supplementary.pdf]

Neural Effects of Creative Movement, General Movement, and Sedentary Play Interventions on Interpersonal Synchrony in Children with Autism Spectrum Disorder: A Preliminary fNIRS Study

Su, W. C.,[1,2], Tsuzuki, D.[3], Srinivasan, S.[4,5,6], Bhat, A. N.[1,2,7*]

Supplemental Table S1. Conditions, examples of training activities, and skills promoted during exemplar CM, GM, and SP intervention sessions.

CONDITION	EXAMPLE ACTIVITIES	TARGETED MOTOR SKILLS	OTHER SKILLS
**CREATIVE MOVEMENT (CM) GROUP – EXEMPLAR SESSION**			
**A. HELLO GAME**	Hot potato: Pass the bean bag around when the music is on, when the music stops, the person who has the bean bag answers a question asked by another.	Bimanual coordination, sensorimotor integration	Social communication, executive functioning
**B. ACTION GAME**	Peter Pan: Sing and play hand games/action sings with partners.	Bimanual coordination, sensorimotor integration, imitation, interpersonal synchrony	Social communication
**C. WARM-UP**	Birthday party: Stretch and use different props (i.e., pool noodles, scarves) to provide sensory inputs on different body parts, e.g., tapping, brushing, etc.	Multi-limb coordination, flexibility, movement improvisation with prompts	Sensory processing
**D. MUSIC TIME**	Stop and Go: Sing and perform whole-body movements with musical instruments (i.e., Maracas, cymbals).<br>Drum circle: Drum synchronously and/or play call and response games (requires turn taking) with the trainer or model.	Whole body and bimanual coordination, imitation, interpersonal synchrony	Executive functioning, social communication
**E. MOVING GAME**	Move and Freeze: When the music starts, move around using one of the locomotor skills. When the music stops, freeze on a yoga spot and maintain balancing poses.	Locomotor skills (i.e., running, galloping, hopping, leaping, jumping, sliding, skipping), balance, strength and endurance, imitation, IPS	Executive functioning, social communication
**F. YOGA AND BREATH**	Yoga story: Do yoga pose according to a storyline (e.g., Luke's beach day). Bird breath: Breathing in and out	Balance, strength and endurance, flexibility, imitation, interpersonal synchrony	Mindfulness, reading, social communication

	while moving arms up and down.		
**G. FAREWELL SONG**	So long, farewell: Sing, reflect, and say goodbye.	Movement improvisation	Social communication
**GENERAL MOTOR (GM) GROUP – EXEMPLAR SESSION**			
**A. HELLO GAME**	Dice Game: Say hello, roll the dice, perform the movement shown, and ask the next player a question.	Motor coordination	Greetings, socially directed attention communication, and turn taking
**B. WARM-UP**	Dynamic Stretches: Perform twist and reach, standing sidekicks, and lateral lunge, etc.	Flexibility, gross motor coordination, and imitation	Socially directed attention, communication, and turn taking
**C. GROW STRONG**	Strength Exercises: Make weighted arm circles, superman reach (prone spinal extension), and superman kicks (prone hip extension).	Upper and lower body strength, complex movement sequences, bilateral and unilateral coordination	Socially directed attention, communication, and turn taking
**D. SPEED UP**	Wheelbarrow Cleanup: Sort balls and beanbags into buckets while sustaining various body positions such as cat position, kneeling, plank, etc.<br>Kick and Toss Combo: Play relay race combining various locomotor skills ending with a kick or throw of a ball at a target.	Locomotor skills, object control skills, visuomotor skills (catching, throwing, targeting), and strength.	Executive Functioning Skills (task shifting, working memory, response inhibition), Socially directed attention, communication, and turn taking.
**E. COOL DOWN**	Static Stretches: Perform lateral neck stretches, standing toe touch, supine spinal twist.	Flexibility, and gross motor coordination	Socially directed attention, communication, and turn taking.
**F. BREATHING**	Deep Breathing Practice: Perform arm breath (arms lift while inhaling and lowering while exhaling).	N/A	Mindfulness, sensory modulation, socially directed attention, communication, and turn taking.
**G. GOODBYE**	Cleanup & Farewell: Reflect questions about favorite exercises/games, cleanup the supplies.	N/A	Prosocial behaviors(helping), socially directed attention, communication, and turn taking.
**SEDENTARY PLAY (SP) GROUP – EXEMPLAR SESSION**			
**A. HELLO**	The Wheel Game: Spin the wheel with the child's and the trainer's/caregiver's names. The person that the arrow lands on gets to answer a question.	N/A	Social communication (includes turn taking, asking and answering questions, natural conversations)

**B. READING**	Story book reading: A picture book is chosen based on the child's reading level and interest. The child is encouraged to read/ attend to the book and answer questions based on the book's content.	N/A	Joint attention, social communication (includes turn taking, asking and answering questions, natural conversations)
**C. WARM-UP**	Peg-boarding: Create a pattern with pegs on the pegboard based on an instruction sheet.	Fine motor coordination/ integration (i.e., peg boarding, lacing, maze drawing)	Social communication (includes turn taking, asking and answering questions, natural conversations)
**D. BUILDING**	LEGO: Build a LEGO creation using pieces according to an instruction sheet<br>Playdoh: Create Playdoh figures based on an instruction sheet<br>The child is encouraged to mimic the trainer's movements during building activities (i.e., pinching, pressing LEGO pieces) and show their creation at each step	Fine motor coordination/ integration (i.e., hold and press LEGO pieces together; flatten, roll, pinch Play-doh)	Joint attention, imitation, social communication (i includes turn taking, asking and answering questions, natural conversations)
**E. FREE BUILD**	Free build: The child is encouraged to build a creation of their choice and talk about their creation.	Movement improvisation, Fine motor coordination	Joint attention, creative play, social communication
**F. ART-CRAFT**	Create a character/scene: Draw, color, cut out, paste, and laminate a character/scene.	Fine motor coordination/ integration (i.e., Coloring, cutting, folding, pasting, handwriting)	Joint attention, imitation, free art, social communication
**G. FAREWELL**	Clean-up: Clean up the workspace and supplies.		Daily living sills (i.e., organization) social communication (includes social elements stated above and reflection on the session)

Supplementary Table S2. Assignment of channels to regions based on spatial registration.

Side	CH	MNI's coordinate			MFG	IFG	STS		IPL			Other			Assigned region
		X	Y	Z	Middle frontal gyrus	Inferior frontal gyrus	Superior temporal gyrus	Middle temporal gyrus	Post-central gyrus	Supramarginal gyrus	Angular gyrus	Superior frontal gyrus	Orbitofrontal gyrus	Precentral gyrus	
Left	7	-33.7	34.3	48.7	96.4							3.6			MFG
	8	-50.0	14.0	47.0	79.5									20.5	Excluded
	9	-60.7	-12.3	45.3					80.2	4.7				15.1	IPL
	10	-60.0	-38.3	45.3						96.2	3.8				IPL
	17	-25.3	53.3	37.3	98.9							1.1			MFG
	18	-47.3	33.3	35.3	100.0										MFG
	19	-60.3	7.0	34.3	4.1				4.9					91.0	Excluded
	20	-67.0	-21.3	33.7					15.4	84.6					IPL
	21	-64.7	-49.3	34.7						54.5	45.5				IPL
	27	-13.7	66.0	26.7	60.1							39.9			MFG
	28	-41.0	52.0	24.7	100.0										MFG
	29	-56.7	26.3	22.7	16.4	83.6									IFG
	30	-66.7	-4.0	22.0					72.3	1.6				26.1	IPL
	31	-69.0	-33.3	21.7			57.4	0.2		42.4					STS
	38	-29.0	66.3	11.3	100.0										MFG
	39	-50.7	44.3	9.7	12.6	87.4									IFG
	40	-60.0	14.7	9.3		60.7								39.3	Excluded
	41	-68.3	-15.3	6.7			69.8	10.5	19.7						STS
	42	-69.7	-44.0	7.3			43.0	57.0							STS
	48	-13.7	73.0	-2.3	63.7							36.0	0.3		MFG
	49	-41.7	59.3	-3.3	21.0	71.1							7.8		IFG
	50	-55.0	33.3	-4.3		69.3	2.6						28.2		IFG
	51	-63.7	1.3	-7.3			76.4	23.6							STS
	52	-71.0	-25.7	-8.7			0.5	99.5							STS
Center	5	16.3	49.3	48.7	17.0							83.0			Excluded
	6	-10.0	49.0	50.0	0.6							99.4			Excluded
	16	4.3	59.7	36.7	13.7							86.3			Excluded

	37	4.3	70.7	11.7	19.9							80.1			Excluded
**Right**	1	66.0	-42.3	41.7						54.4	45.6				IPL
	2	65.7	-16.7	42.7					11.6	88.4					IPL
	3	55.3	11.3	44.3	35.8									64.2	Exclude
	4	39.7	34.7	46.7	100.0										MFG
	11	65.0	-53.3	29.7			8.2	0.6		5.6	85.7				IPL
	12	70.7	-26.7	30.7					2.2	93.1	4.7				IPL
	13	66.0	2.7	30.3					51.0					49.0	Excluded
	14	53.0	31.7	32.7	67.3	32.7									MFG
	15	31.7	52.7	35.7	100.0										MFG
	22	71.0	-39.3	16.7			72.9	17.3		4.8	5.0				STS
	23	70.0	-8.7	17.3			20.2		76.5	3.3					IPL
	24	61.3	23.7	18.3		56.7								43.3	IFG
	25	45.7	51.3	21.7	63.5	36.5									MFG
	26	20.3	66.3	25.7	97.5							2.5			MFG
	32	69.7	-51.0	3.0			99.5	0.5							STS
	33	73.0	-21.7	1.7			59.1	40.9							STS
	34	63.3	9.7	5.7		5.9	34.8		4.9					54.3	Excluded
	35	55.0	42.3	6.7	2.1	97.9									IFG
	36	34.3	65.7	10.0	96.1	3.9									MFG
	43	72.0	-33.7	-11.7			5.5	94.5							STS
	44	69.0	-4.7	-11.3			23.5	76.5							STS
	45	57.0	29.3	-5.7		85.9	3.4						10.7		IFG
	46	46.3	57.0	-5.3		83.6							16.4		IFG
	47	19.3	72.0	-3.3	96.5							1.0	2.4		MFG

For each channel, the spatial location in MNI's coordinate system and the probability of covering different brain regions were shown. The channels were then assigned to a specific ROI.

Supplementary Table S3. Means and standard errors of synchrony performance in children in CM, GM, and SP groups.

Group activation data	Pre-test		Post-test	
	**Mean**	**SE**	**Mean**	**SE**
**CM group**				
Solo condition				
Rhythmic error	0.34	0.14	0.22	0.15
Social condition				
Rhythmic error	0.7	0.09	0.33	0.08
Synchrony error	1.18	0.17	0.64	0.24
Mirroring error	1.21	0.17	0.76	0.23
**GM group**				
Solo condition				
Rhythmic error	0.75	0.13	0.5	0.14
Social condition				
Rhythmic error	0.71	0.10	0.61	0.17
Synchrony error	1.18	0.21	0.68	0.20
Mirroring error	1.54	0.15	1.18	0.22
**SP group**				
Solo condition				
Rhythmic error	0.38	0.20	0.50	0.19
Social condition				
Rhythmic error	0.79	0.11	0.61	0.19
Synchrony error	0.96	0.19	0.86	0.27
Mirroring error	1.57	0.24	1.36	0.29

Supplementary Table S4. Summary of t-test analyses, effect sizes, and the individual trends for the interpersonal synchrony performance.

Comparison	*p*-values	Direction of effect	Effect sizes (r)	% of children showing the trend
**Time related difference in error #**				
CM, Social, Rhythm error	0.021	Pre > Post *	-0.82	87.5
CM, Social, Synchrony error	0.042	Pre > Post *	-0.72	87.5
CM, Social Mirroring error	0.041	Pre > Post *	-0.72	75.0

* *p*-values < 0.05 which did not survive FDR correction.

Supplementary Table S5. Summary of the Bayesian ANCOVA analyses

Models	BFM	BF10	error %
**Null model (incl. Age, SUBJECT, Method)**	8.977	1	
**time**	64.869	5.477	3.528
**time + hemisphere**	37.756	3.612	4.703
**time + hemisphere + time ✻ hemisphere**	22.827	2.356	3.915
**time + region**	14.966	1.612	2.444
**time + hemisphere + region**	10.466	1.156	6.065

**time + hemisphere + region + time ✻ hemisphere**	6.831	0.77	4.846
**Group + time**	6.314	0.714	6.032
**hemisphere**	5.692	0.646	1.874
**Group + time + hemisphere**	3.621	0.416	2.54
**region**	2.581	0.298	0.96
**Group + time + hemisphere + time ✻ hemisphere**	2.501	0.289	3.5
**Group + time + region**	1.752	0.204	2.29
**hemisphere + region**	1.618	0.188	2.002
**Group + time + hemisphere + region**	1.135	0.132	3.967
**Group**	1.092	0.127	1.339
**Group + time + hemisphere + region + time ✻ hemisphere**	0.895	0.104	5.164
**Group + hemisphere**	0.701	0.082	2.424
**Group + time + Group ✻ time**	0.456	0.053	2.108
**Group + region**	0.33	0.039	1.902
**Group + time + hemisphere + Group ✻ time**	0.284	0.033	2.161
**Group + hemisphere + region**	0.202	0.024	1.539
**Group + time + hemisphere + Group ✻ time + time ✻ hemisphere**	0.195	0.023	3.261
**time + region + time ✻ region**	0.181	0.021	5.875
**Group + time + region + Group ✻ time**	0.151	0.018	5.431
**time + hemisphere + region + time ✻ region**	0.122	0.014	9.429
**time + hemisphere + region + time ✻ hemisphere + time ✻ region**	0.092	0.011	10.458
**Group + time + hemisphere + region + Group ✻ time**	0.089	0.01	2.674
**time + hemisphere + region + hemisphere ✻ region**	0.084	0.01	3.682
**Group + time + hemisphere + region + Group ✻ time + time ✻ hemisphere**	0.07	0.008	7.246
**Group + time + hemisphere + Group ✻ hemisphere**	0.064	0.008	2.153
**time + hemisphere + region + time ✻ hemisphere + hemisphere ✻ region**	0.054	0.006	2.006
**Group + time + hemisphere + Group ✻ hemisphere + time ✻ hemisphere**	0.047	0.006	3.778
**Group + time + region + time ✻ region**	0.022	0.003	2.451
**Group + time + hemisphere + region + Group ✻ hemisphere**	0.021	0.002	3.314
**Group + time + hemisphere + region + Group ✻ time + time ✻ region**	0.019	0.002	93.365
**Group + time + hemisphere + region + Group ✻ hemisphere + time ✻ hemisphere**	0.015	0.002	7.211
**hemisphere + region + hemisphere ✻ region**	0.015	0.002	1.458
**Group + time + hemisphere + region + time ✻ region**	0.015	0.002	3.824
**Group + hemisphere + Group ✻ hemisphere**	0.012	0.001	3.214
**Group + time + hemisphere + region + hemisphere ✻ region**	0.012	0.001	5.539
**Group + time + hemisphere + region + time ✻ hemisphere + time ✻ region**	0.011	0.001	6.457
**Group + time + hemisphere + region + time ✻ hemisphere + hemisphere ✻ region**	0.008	9.087×10-4	4.154
**Group + time + hemisphere + Group ✻ time + Group ✻ hemisphere**	0.006	7.504×10-4	9.385
**Group + time + region + Group ✻ region**	0.006	7.221×10-4	6.279
**Group + time + hemisphere + Group ✻ time + Group ✻ hemisphere + time ✻ hemisphere**	0.005	5.810×10-4	22.682
**Group + time + hemisphere + region + Group ✻ region**	0.004	4.520×10-4	2.766
**Group + hemisphere + region + Group ✻ hemisphere**	0.004	4.275×10-4	2.208
**Group + time + hemisphere + region + time ✻ hemisphere + Group ✻ region**	0.003	3.144×10-4	3.16
**Group + hemisphere + region + hemisphere ✻ region**	0.002	2.326×10-4	4.764

Group + time + region + Group ∗ time + time ∗ region	0.002	2.106×10-4	2.925
Group + time + hemisphere + region + Group ∗ time + Group ∗ hemisphere	0.002	1.927×10-4	3.288
time + hemisphere + region + time ∗ region + hemisphere ∗ region	0.001	1.553×10-4	19.168
Group + time + hemisphere + region + Group ∗ time + Group ∗ hemisphere + time ∗ hemisphere	0.001	1.402×10-4	4.223
Group + region + Group ∗ region	0.001	1.223×10-4	1.542
Group + time + hemisphere + region + Group ∗ time + hemisphere ∗ region	8.743×10-4	1.027×10-4	3.926
Group + time + hemisphere + region + Group ∗ time + time ∗ hemisphere + time ∗ region	8.530×10-4	1.002×10-4	4.548
time + hemisphere + region + time ∗ hemisphere + time ∗ region + hemisphere ∗ region	7.777×10-4	9.133×10-5	5.47
Group + hemisphere + region + Group ∗ region	6.880×10-4	8.078×10-5	2.808
Group + time + hemisphere + region + Group ∗ time + time ∗ hemisphere + hemisphere ∗ region	6.128×10-4	7.196×10-5	3.682
Group + time + region + Group ∗ time + Group ∗ region	4.813×10-4	5.652×10-5	2.268
Group + time + hemisphere + region + Group ∗ time + Group ∗ region	3.043×10-4	3.573×10-5	2.867
Group + time + hemisphere + region + Group ∗ hemisphere + time ∗ region	2.971×10-4	3.488×10-5	6.261
Group + time + hemisphere + Group ∗ time + Group ∗ hemisphere + time ∗ hemisphere + Group ∗ time ∗ hemisphere	2.953×10-4	3.468×10-5	7.097
Group + time + hemisphere + region + Group ∗ hemisphere + time ∗ hemisphere + time ∗ region	2.122×10-4	2.492×10-5	11.876
Group + time + hemisphere + region + Group ∗ time + time ∗ hemisphere + Group ∗ region	2.018×10-4	2.370×10-5	2.415
Group + time + hemisphere + region + Group ∗ hemisphere + hemisphere ∗ region	1.873×10-4	2.200×10-5	2.69
Group + time + hemisphere + region + Group ∗ hemisphere + time ∗ hemisphere + hemisphere ∗ region	1.405×10-4	1.650×10-5	9.189
Group + time + hemisphere + region + time ∗ region + hemisphere ∗ region	1.355×10-4	1.591×10-5	2.752
Group + time + hemisphere + region + time ∗ hemisphere + time ∗ region + hemisphere ∗ region	9.739×10-5	1.144×10-5	3.918
Group + time + hemisphere + region + Group ∗ time + Group ∗ hemisphere + time ∗ hemisphere + Group ∗ time ∗ hemisphere	8.853×10-5	1.040×10-5	3.569
Group + time + region + Group ∗ region + time ∗ region	7.772×10-5	9.126×10-6	3.505
Group + time + hemisphere + region + Group ∗ hemisphere + Group ∗ region	7.735×10-5	9.083×10-6	6.597
Group + time + hemisphere + region + Group ∗ hemisphere + time ∗ hemisphere + Group ∗ region	5.544×10-5	6.510×10-6	8.922
Group + time + hemisphere + region + Group ∗ region + time ∗ region	4.715×10-5	5.537×10-6	2.138
Group + time + hemisphere + region + time ∗ hemisphere + Group ∗ region + time ∗ region	4.008×10-5	4.707×10-6	10.106
Group + hemisphere + region + Group ∗ hemisphere + hemisphere ∗ region	3.650×10-5	4.286×10-6	3.979
Group + time + hemisphere + region + Group ∗ region + hemisphere ∗ region	3.513×10-5	4.125×10-6	3.633
Group + time + hemisphere + region + time ∗ hemisphere + Group ∗ region + hemisphere ∗ region	2.481×10-5	2.913×10-6	3.269
Group + time + hemisphere + region + Group ∗ time + Group ∗ hemisphere + time ∗ region	2.154×10-5	2.529×10-6	3.384

time + hemisphere + region + time ✻ hemisphere + time ✻ region + hemisphere ✻ region + time ✻ hemisphere ✻ region	2.068×10-5	2.428×10-6	2.783
Group + time + hemisphere + region + Group ✻ time + Group ✻ hemisphere + time ✻ hemisphere + time ✻ region	1.916×10-5	2.250×10-6	15.78
Group + time + hemisphere + region + Group ✻ time + Group ✻ hemisphere + hemisphere ✻ region	1.559×10-5	1.831×10-6	4.03
Group + hemisphere + region + Group ✻ hemisphere + Group ✻ region	1.298×10-5	1.524×10-6	4.287
Group + time + hemisphere + region + Group ✻ time + Group ✻ hemisphere + time ✻ hemisphere + hemisphere ✻ region	1.267×10-5	1.488×10-6	11.75
Group + time + hemisphere + region + Group ✻ time + time ✻ region + hemisphere ✻ region	1.124×10-5	1.320×10-6	4.311
Group + time + hemisphere + region + Group ✻ time + time ✻ hemisphere + time ✻ region + hemisphere ✻ region	7.957×10-6	9.344×10-7	4.446
Group + time + region + Group ✻ time + Group ✻ region + time ✻ region	6.621×10-6	7.775×10-7	7
Group + hemisphere + region + Group ✻ region + hemisphere ✻ region	6.583×10-6	7.730×10-7	3.85
Group + time + hemisphere + region + Group ✻ time + Group ✻ hemisphere + Group ✻ region	5.268×10-6	6.186×10-7	3.02
Group + time + hemisphere + region + Group ✻ time + Group ✻ hemisphere + time ✻ hemisphere + Group ✻ region	3.921×10-6	4.604×10-7	3.704
Group + time + hemisphere + region + Group ✻ time + Group ✻ region + time ✻ region	3.909×10-6	4.591×10-7	3.257
Group + time + hemisphere + region + time ✻ hemisphere + time ✻ region + hemisphere ✻ region + time ✻ hemisphere ✻ region	2.905×10-6	3.411×10-7	7.301
Group + time + hemisphere + region + Group ✻ time + Group ✻ region + hemisphere ✻ region	2.798×10-6	3.286×10-7	3.095
Group + time + hemisphere + region + Group ✻ time + time ✻ hemisphere + Group ✻ region + time ✻ region	2.688×10-6	3.157×10-7	3.949
Group + time + hemisphere + region + Group ✻ hemisphere + time ✻ region + hemisphere ✻ region	2.466×10-6	2.896×10-7	3.73
Group + time + hemisphere + region + Group ✻ time + time ✻ hemisphere + Group ✻ region + hemisphere ✻ region	2.056×10-6	2.415×10-7	4.351
Group + time + hemisphere + region + Group ✻ hemisphere + time ✻ hemisphere + time ✻ region + hemisphere ✻ region	1.734×10-6	2.037×10-7	5.867
Group + time + hemisphere + region + Group ✻ time + Group ✻ hemisphere + time ✻ hemisphere + time ✻ region + Group ✻ time ✻ hemisphere	1.411×10-6	1.657×10-7	18.528
Group + time + hemisphere + region + Group ✻ hemisphere + Group ✻ region + time ✻ region	9.308×10-7	1.093×10-7	4.554
Group + time + hemisphere + region + Group ✻ time + Group ✻ hemisphere + time ✻ hemisphere + hemisphere ✻ region + Group ✻ time ✻ hemisphere	8.606×10-7	1.011×10-7	4.594
Group + time + hemisphere + region + Group ✻ hemisphere + Group ✻ region + hemisphere ✻ region	6.783×10-7	7.965×10-8	3.278
Group + time + hemisphere + region + Group ✻ hemisphere + time ✻ hemisphere + Group ✻ region + time ✻ region	6.068×10-7	7.125×10-8	3.681
Group + time + hemisphere + region + Group ✻ hemisphere + time ✻ hemisphere + Group ✻ region + hemisphere ✻ region	4.937×10-7	5.797×10-8	7.346
Group + time + hemisphere + region + Group ✻ region + time ✻ region + hemisphere ✻ region	4.656×10-7	5.467×10-8	5.058
Group + time + hemisphere + region + Group ✻ time + Group ✻ hemisphere + time ✻ hemisphere + Group ✻ region + Group ✻ time ✻ hemisphere	4.170×10-7	4.897×10-8	17.159
Group + time + hemisphere + region + time ✻ hemisphere + Group ✻ region + time ✻ region + hemisphere ✻ region	3.347×10-7	3.931×10-8	7.66

**Group + time + hemisphere + region + Group ✻ time + time ✻ hemisphere + time ✻ region + hemisphere ✻ region + time ✻ hemisphere ✻ region**	2.174×10-7	2.553×10-8	3.872
**Group + time + hemisphere + region + Group ✻ time + Group ✻ hemisphere + time ✻ region + hemisphere ✻ region**	2.005×10-7	2.354×10-8	4.167
**Group + time + hemisphere + region + Group ✻ time + Group ✻ hemisphere + time ✻ hemisphere + time ✻ region + hemisphere ✻ region**	1.442×10-7	1.694×10-8	6.395
**Group + hemisphere + region + Group ✻ hemisphere + Group ✻ region + hemisphere ✻ region**	1.120×10-7	1.316×10-8	2.1
**Group + time + hemisphere + region + Group ✻ time + Group ✻ hemisphere + Group ✻ region + hemisphere ✻ region**	1.014×10-7	1.191×10-8	45.509
**Group + time + region + Group ✻ time + Group ✻ region + time ✻ region + Group ✻ time ✻ region**	8.648×10-8	1.016×10-8	2.477
**Group + time + hemisphere + region + Group ✻ time + Group ✻ hemisphere + Group ✻ region + time ✻ region**	7.233×10-8	8.494×10-9	3.964
**Group + time + hemisphere + region + Group ✻ time + Group ✻ region + time ✻ region + Group ✻ time ✻ region**	6.075×10-8	7.134×10-9	4.534
**Group + time + hemisphere + region + Group ✻ time + Group ✻ hemisphere + time ✻ hemisphere + Group ✻ region + time ✻ region**	5.278×10-8	6.197×10-9	6.356
**Group + time + hemisphere + region + Group ✻ hemisphere + time ✻ hemisphere + time ✻ region + hemisphere ✻ region + time ✻ hemisphere ✻ region**	5.072×10-8	5.956×10-9	3.891
**Group + time + hemisphere + region + Group ✻ time + Group ✻ region + time ✻ region + hemisphere ✻ region**	4.898×10-8	5.751×10-9	17.733
**Group + time + hemisphere + region + Group ✻ time + time ✻ hemisphere + Group ✻ region + time ✻ region + Group ✻ time ✻ region**	4.033×10-8	4.736×10-9	3.482
**Group + time + hemisphere + region + Group ✻ time + Group ✻ hemisphere + time ✻ hemisphere + Group ✻ region + hemisphere ✻ region**	3.658×10-8	4.295×10-9	4.202
**Group + time + hemisphere + region + Group ✻ time + time ✻ hemisphere + Group ✻ region + time ✻ region + hemisphere ✻ region**	2.537×10-8	2.979×10-9	3.504
**Group + time + hemisphere + region + time ✻ hemisphere + Group ✻ region + time ✻ region + hemisphere ✻ region + time ✻ hemisphere ✻ region**	1.233×10-8	1.448×10-9	22.494
**Group + time + hemisphere + region + Group ✻ time + Group ✻ hemisphere + time ✻ hemisphere + time ✻ region + hemisphere ✻ region + Group ✻ time ✻ hemisphere**	1.085×10-8	1.274×10-9	4.902
**Group + time + hemisphere + region + Group ✻ hemisphere + Group ✻ region + time ✻ region + hemisphere ✻ region**	8.942×10-9	1.050×10-9	12.362
**Group + time + hemisphere + region + Group ✻ hemisphere + time ✻ hemisphere + Group ✻ region + time ✻ region + hemisphere ✻ region**	5.659×10-9	6.645×10-10	3.545
**Group + time + hemisphere + region + Group ✻ time + Group ✻ hemisphere + time ✻ hemisphere + time ✻ region + hemisphere ✻ region + time ✻ hemisphere ✻ region**	3.866×10-9	4.540×10-10	4.215
**Group + time + hemisphere + region + Group ✻ time + Group ✻ hemisphere + time ✻ hemisphere + Group ✻ region + time ✻ region + Group ✻ time ✻ hemisphere**	3.778×10-9	4.436×10-10	3.568
**Group + time + hemisphere + region + Group ✻ time + Group ✻ hemisphere + time ✻ hemisphere + Group ✻ region + hemisphere ✻ region + Group ✻ time ✻ hemisphere**	3.375×10-9	3.963×10-10	8.684
**Group + time + hemisphere + region + Group ✻ hemisphere + Group ✻ region + hemisphere ✻ region + Group ✻ hemisphere ✻ region**	1.806×10-9	2.121×10-10	7.418
**Group + time + hemisphere + region + Group ✻ hemisphere + time ✻ hemisphere + Group ✻ region + hemisphere ✻ region + Group ✻ hemisphere ✻ region**	1.222×10-9	1.434×10-10	7.655
**Group + time + hemisphere + region + Group ✻ time + Group ✻ hemisphere + Group ✻ region + time ✻ region + Group ✻ time ✻ region**	1.152×10-9	1.352×10-10	4.589
**Group + time + hemisphere + region + Group ✻ time + Group ✻ region + time ✻ region + hemisphere ✻ region + Group ✻ time ✻ region**	7.958×10-10	9.344×10-11	31.287

**Group + time + hemisphere + region + Group ∗ time + time ∗ hemisphere + Group ∗ region + time ∗ region + hemisphere ∗ region + time ∗ hemisphere ∗ region**	7.621×10-10	8.949×10-11	4.547
**Group + time + hemisphere + region + Group ∗ time + Group ∗ hemisphere + time ∗ hemisphere + Group ∗ region + time ∗ region + Group ∗ time ∗ region**	7.598×10-10	8.922×10-11	4.844
**Group + time + hemisphere + region + Group ∗ time + Group ∗ hemisphere + Group ∗ region + time ∗ region + hemisphere ∗ region**	6.514×10-10	7.649×10-11	3.394
**Group + time + hemisphere + region + Group ∗ time + Group ∗ hemisphere + time ∗ hemisphere + Group ∗ region + time ∗ region + hemisphere ∗ region**	5.074×10-10	5.958×10-11	10.439
**Group + time + hemisphere + region + Group ∗ time + time ∗ hemisphere + Group ∗ region + time ∗ region + hemisphere ∗ region + Group ∗ time ∗ region**	4.139×10-10	4.860×10-11	5.814
**Group + hemisphere + region + Group ∗ hemisphere + Group ∗ region + hemisphere ∗ region + Group ∗ hemisphere ∗ region**	3.404×10-10	3.997×10-11	6.071
**Group + time + hemisphere + region + Group ∗ time + Group ∗ hemisphere + time ∗ hemisphere + time ∗ region + hemisphere ∗ region + Group ∗ time ∗ hemisphere + time ∗ hemisphere ∗ region**	3.046×10-10	3.576×10-11	5.152
**Group + time + hemisphere + region + Group ∗ hemisphere + time ∗ hemisphere + Group ∗ region + time ∗ region + hemisphere ∗ region + time ∗ hemisphere ∗ region**	1.856×10-10	2.179×10-11	6.781
**Group + time + hemisphere + region + Group ∗ time + Group ∗ hemisphere + Group ∗ region + hemisphere ∗ region + Group ∗ hemisphere ∗ region**	1.367×10-10	1.605×10-11	3.354
**Group + time + hemisphere + region + Group ∗ time + Group ∗ hemisphere + time ∗ hemisphere + Group ∗ region + hemisphere ∗ region + Group ∗ hemisphere ∗ region**	1.082×10-10	1.270×10-11	5.841
**Group + time + hemisphere + region + Group ∗ time + Group ∗ hemisphere + time ∗ hemisphere + Group ∗ region + time ∗ region + Group ∗ time ∗ hemisphere + Group ∗ time ∗ region**	5.950×10-11	6.987×10-12	4.245
**Group + time + hemisphere + region + Group ∗ time + Group ∗ hemisphere + time ∗ hemisphere + Group ∗ region + time ∗ region + hemisphere ∗ region + Group ∗ time ∗ hemisphere**	3.889×10-11	4.566×10-12	6.289
**Group + time + hemisphere + region + Group ∗ hemisphere + Group ∗ region + time ∗ region + hemisphere ∗ region + Group ∗ hemisphere ∗ region**	2.141×10-11	2.515×10-12	3.104
**Group + time + hemisphere + region + Group ∗ hemisphere + time ∗ hemisphere + Group ∗ region + time ∗ region + hemisphere ∗ region + Group ∗ hemisphere ∗ region**	1.591×10-11	1.868×10-12	5.088
**Group + time + hemisphere + region + Group ∗ time + Group ∗ hemisphere + time ∗ hemisphere + Group ∗ region + time ∗ region + hemisphere ∗ region + time ∗ hemisphere ∗ region**	1.353×10-11	1.588×10-12	5.261
**Group + time + hemisphere + region + Group ∗ time + time ∗ hemisphere + Group ∗ region + time ∗ region + hemisphere ∗ region + Group ∗ time ∗ region + time ∗ hemisphere ∗ region**	1.243×10-11	1.459×10-12	11.554
**Group + time + hemisphere + region + Group ∗ time + Group ∗ hemisphere + Group ∗ region + time ∗ region + hemisphere ∗ region + Group ∗ time ∗ region**	1.110×10-11	1.303×10-12	6.99
**Group + time + hemisphere + region + Group ∗ time + Group ∗ hemisphere + time ∗ hemisphere + Group ∗ region + hemisphere ∗ region + Group ∗ time ∗ hemisphere + Group ∗ hemisphere ∗ region**	7.789×10-12	9.146×10-13	3.968
**Group + time + hemisphere + region + Group ∗ time + Group ∗ hemisphere + time ∗ hemisphere + Group ∗ region + time ∗ region + hemisphere ∗ region + Group ∗ time ∗ region**	7.779×10-12	9.134×10-13	4.92
**Group + time + hemisphere + region + Group ∗ time + Group ∗ hemisphere + Group ∗ region + time ∗ region + hemisphere ∗ region + Group ∗ hemisphere ∗ region**	1.816×10-12	2.133×10-13	4.013

**Group + time + hemisphere + region + Group ✻ time + Group ✻ hemisphere + time ✻ hemisphere + Group ✻ region + time ✻ region + hemisphere ✻ region + Group ✻ hemisphere ✻ region**	1.248×10-12	1.465×10-13	3.603
**Group + time + hemisphere + region + Group ✻ time + Group ✻ hemisphere + time ✻ hemisphere + Group ✻ region + time ✻ region + hemisphere ✻ region + Group ✻ time ✻ hemisphere + Group ✻ time ✻ region**	5.672×10-13	6.660×10-14	8.134
**Group + time + hemisphere + region + Group ✻ hemisphere + time ✻ hemisphere + Group ✻ region + time ✻ region + hemisphere ✻ region + Group ✻ hemisphere ✻ region + time ✻ hemisphere ✻ region**	4.771×10-13	5.603×10-14	5.009
**Group + time + hemisphere + region + Group ✻ time + Group ✻ hemisphere + time ✻ hemisphere + Group ✻ region + time ✻ region + hemisphere ✻ region + Group ✻ time ✻ region + time ✻ hemisphere ✻ region**	2.177×10-13	2.556×10-14	6.092
**Group + time + hemisphere + region + Group ✻ time + Group ✻ hemisphere + time ✻ hemisphere + Group ✻ region + time ✻ region + hemisphere ✻ region + Group ✻ time ✻ hemisphere + Group ✻ hemisphere ✻ region**	1.124×10-13	1.319×10-14	8.817
**Group + time + hemisphere + region + Group ✻ time + Group ✻ hemisphere + Group ✻ region + time ✻ region + hemisphere ✻ region + Group ✻ time ✻ region + Group ✻ hemisphere ✻ region**	4.212×10-14	4.946×10-15	31.171
**Group + time + hemisphere + region + Group ✻ time + Group ✻ hemisphere + time ✻ hemisphere + Group ✻ region + time ✻ region + hemisphere ✻ region + Group ✻ hemisphere ✻ region + time ✻ hemisphere ✻ region**	4.027×10-14	4.729×10-15	5.273
**Group + time + hemisphere + region + Group ✻ time + Group ✻ hemisphere + time ✻ hemisphere + Group ✻ region + time ✻ region + hemisphere ✻ region + Group ✻ time ✻ region + Group ✻ hemisphere ✻ region**	2.140×10-14	2.513×10-15	10.214
**Group + time + hemisphere + region + Group ✻ time + Group ✻ hemisphere + time ✻ hemisphere + Group ✻ region + time ✻ region + hemisphere ✻ region + Group ✻ time ✻ hemisphere + time ✻ hemisphere ✻ region**	1.754×10-14	2.059×10-15	59.331
**Group + time + hemisphere + region + Group ✻ time + Group ✻ hemisphere + time ✻ hemisphere + Group ✻ region + time ✻ region + hemisphere ✻ region + Group ✻ time ✻ hemisphere + Group ✻ time ✻ region + time ✻ hemisphere ✻ region**	1.702×10-14	1.999×10-15	6.853
**Group + time + hemisphere + region + Group ✻ time + Group ✻ hemisphere + time ✻ hemisphere + Group ✻ region + time ✻ region + hemisphere ✻ region + Group ✻ time ✻ hemisphere + Group ✻ hemisphere ✻ region + time ✻ hemisphere ✻ region**	2.694×10-15	3.163×10-16	6.18
**Group + time + hemisphere + region + Group ✻ time + Group ✻ hemisphere + time ✻ hemisphere + Group ✻ region + time ✻ region + hemisphere ✻ region + Group ✻ time ✻ hemisphere + Group ✻ time ✻ region + Group ✻ hemisphere ✻ region**	1.499×10-15	1.761×10-16	5.067
**Group + time + hemisphere + region + Group ✻ time + Group ✻ hemisphere + time ✻ hemisphere + Group ✻ region + time ✻ region + hemisphere ✻ region + Group ✻ time ✻ region + Group ✻ hemisphere ✻ region + time ✻ hemisphere ✻ region**	5.966×10-16	7.006×10-17	5.739
**Group + time + hemisphere + region + Group ✻ time + Group ✻ hemisphere + time ✻ hemisphere + Group ✻ region + time ✻ region + hemisphere ✻ region + Group ✻ time ✻ hemisphere + Group ✻ time ✻ region + Group ✻ hemisphere ✻ region + time ✻ hemisphere ✻ region**	5.111×10-17	6.001×10-18	10.387
**Group + time + hemisphere + region + Group ✻ time + Group ✻ hemisphere + time ✻ hemisphere + Group ✻ region + time ✻ region + hemisphere ✻ region + Group ✻ time ✻ hemisphere + Group ✻ time ✻ region + Group ✻ hemisphere ✻ region + time ✻ hemisphere ✻ region + Group ✻ time ✻ hemisphere ✻ region**	6.273×10-18	7.366×10-19	6.11

Note. Models = models being compared; BFM = Bayes Factor for the model versus all other models (values > 1 indicate greater support for the model); BF_{10} = Bayes Factor comparing the model to the null

model (values > 1 indicate greater support for the model over the null); Error % = error percentage in estimating the Bayes factor (values < 1% indicate more precise estimation).

Supplementary Table S6. Component matrix for Principal Component Analyses.

ROI	Component 1
Pre-test	
Left MFG	0.686
Left IFG	0.717
Left STS	0.598
Left IPL	0.688
Right MFG	0.740
Right IFG	0.526
Right STS	0.601
Right IPL	0.601
Post-test	
Left MFG	0.095
Left IFG	0.212
Left STS	0.096
Left IPL	0.123
Right MFG	0.116
Right IFG	0.078
Right STS	-0.310
Right IPL	-0.191

Supplementary Table S7. Means and standard errors of Social-Solo HbO_2 concentration in children in CM, GM, and SP groups.

**Group activation data**	**Pre-test**		**Post-test**	
	**Mean**	**SE**	**Mean**	**SE**
**CM group**				
Left hemisphere				
MFG	-0.001	0.011	0.023	0.011
IFG	-0.017	0.018	0.021	0.015
STS	-0.022	0.012	0.030	0.017
IPL	0.011	0.013	0.016	0.021
Right hemisphere				
MFG	0.005	0.011	0.033	0.019
IFG	0.017	0.016	0.030	0.023
STS	-0.006	0.010	0.016	0.013
IPL	0.014	0.013	0.015	0.014
**GM group**				
Left hemisphere				
MFG	0.009	0.014	0.001	0.020
IFG	-0.018	0.018	0.050	0.021
STS	0.015	0.021	-0.031	0.026
IPL	-0.019	0.021	-0.006	0.013
Right hemisphere				
MFG	0.021	0.017	0.008	0.013
IFG	0.036	0.019	0.024	0.020
STS	0.006	0.023	0.017	0.029
IPL	-0.009	0.017	-0.006	0.012
**SP group**				
Left hemisphere				
MFG	0.005	0.024	0.036	0.013
IFG	0.021	0.025	0.058	0.021
STS	-0.043	0.022	0.047	0.021
IPL	0.012	0.023	0.026	0.018
Right hemisphere				
MFG	0.040	0.016	0.048	0.014
IFG	0.053	0.021	0.058	0.020
STS	0.030	0.025	0.022	0.019
IPL	0.018	0.024	0.012	0.019

Supplementary Table S8. Post-hoc analyses of Social – Solo cortical activation for the Group × Time × Hemisphere x Region 4-way interaction.

**Comparison**	***p*-values**	**Direction of effect**	**Effect sizes (Hedge's g)**	**# of children showing the trend**
**Group related difference**				
Pre, Left, STS	0.066	GM > SP↓	0.09 (-0.96 to 1.14)	-
Pre, Right, MFG	0.079	SP > CM↓	0.06 (-0.95 to 1.08)	-
Post, Left, STS	0.055	CM > GM↓	0.09 (-0.92 to 1.11)	-
Post, Left, STS	0.023	SP > GM*	0.12 (-0.93 to 1.17)	-
Post, Right, MFG	0.039	SP > GM*	0.08 (-0.97 to 1.13)	-
**Time related difference**				
CM group, Left, MFG	0.094	Post > Pre↓	0.70 (-0.26 to 1.66)	100.0
CM group, Left, IFG	0.068	Post > Pre↓	0.68 (-0.28 to 1.63)	100.0
CM group, Left, STS	0.007	Post > Pre*	**1.34 (0.08 to 2.60)**	87.5
GM group, Left IFG	0.019	Post > Pre*	1.27 (-0.12 to 2.65)	57.1
SP group, Left, STS	0.008	Post > Pre*	1.32 (-0.10 to 2.74)	85.7
**Hemisphere related difference**				
CM group, Pre, IFG	0.056	Right > Left↓	0.60 (-0.32 to 1.53)	87.5
GM group, Pre, IFG	0.031	Right > Left*	1.02 (-0.22 to 2.25)	71.4
SP group, Pre, MFG	0.097	Right > Left↓	0.48 (-0.51 to 1.47)	57.1
SP group Pre, STS	0.012	Right > Left*	1.07 (-0.20 to 2.33)	71.4

* *p*-values < 0.05 which did not survive FDR correction. ↓ a trend of significant difference, *p*-values between 0.05 and 0.1. Bold font indicates that the 95% CIs of the within and between-group comparisons does not include 0

Supplementary Table S9. Correlation between baseline socially enhanced activation and improvements in IPS performance

To associate baseline socially enhanced activation with intervention-related changes in IPS performance during the drumming task, we correlated improvements in synchrony performance with socially enhanced activation values at the pretest. In the CM group, children exhibiting lower right IFG, STS, and IPL activation at the pretest had more reduction in Solo-rhythm errors (rs = 0.35 to 0.41; *p*s < 0.05), and children exhibiting lower left IFG activation at the pretest had more reduction in Social-mirroring errors (r = 0.36, 95% CI = 0.02 to 0.63; $p < 0.05$; **Table 4**). Similarly, in the GM group, children exhibiting lower left MFG activation during the pretest had greater reduction in Solo-rhythm errors (r = 0.38; 95% CI = 0.02 to 0.66; $p < 0.05$; **Table 4**). Lastly, in the SP group, children exhibiting lower left MFG activation at the pretest had more reduction in Social-synchrony errors (r = 0.55; 95% CI = -0.23 to 0.77; $p < 0.01$; **Table 4**). Overall, autistic children with lower activation at pretest in various OEMS regions had greater intervention-related improvements (i.e., greater reduction in error), post-intervention.

**r-values**	**Solo**	**Social**		
	**Δ Rhythm**	**Δ Rhythm**	**Δ Synchrony**	**Δ Mirror**
CM group				
Left hemisphere				
MFG	0.11 (-0.24 to 0.44)	-0.02 (-0.36 to 0.33)	0.06 (-0.29 to 0.39)	0.11 (-0.24 to 0.44)
IFG	0.30 (-0.05 to 0.58)	0.04 (-0.30 to 0.38)	0.29 (-0.06 to 0.57)	**0.36 (0.02 to 0.63)***
STS	0.15 (-0.21 to 0.47)	0.19 (-0.17 to 0.50)	0.18 (-0.18 to 0.49)	0.01 (-0.34 to 0.35)
IPL	0.28 (-0.07 to 0.57)	-0.22 (-0.53 to 0.13)	-0.01 (-0.36 to 0.33)	-0.04 (-0.11 to 0.54)
Right hemisphere				

MFG	0.23 (-0.13 to 0.53)	0.06 (-0.29 to 0.39)	0.22 (-0.13 to 0.53)	0.24 (-0.11 to 0.54)
IFG	**0.41 (0.08 to 0.66)***	0.10 (-0.26 to 0.43)	0.19 (-0.17 to 0.50)	0.21 (-0.15 to 0.51)
STS	**0.36 (0.02 to 0.63)***	0.01 (-0.34 to 0.35)	0.11 (-0.25 to 0.43)	0.23 (-0.13 to 0.53)
IPL	**0.35 (0.00 to 0.62)***	-0.06 (-0.39 to 0.29)	-0.16 (-0.48 to 0.20)	-0.10 (-0.43 to 0.25)
GM group				
Left hemisphere				
MFG	**0.38 (0.02 to 0.66)***	0.10 (-0.28 to 0.45)	-0.01 (-0.38 to 0.36)	0.20 (-0.18 to 0.53)
IFG	0.22 (-0.16 to 0.55)	-0.33 (-0.62 to 0.04)	-0.05 (-0.41 to 0.33)	-0.19 (-0.52 to 0.19)
STS	0.27 (-0.11 to 0.58)	-0.18 (-0.51 to 0.20)	0.02 (-0.35 to 0.38)	-0.09 (-0.44 to 0.29)
IPL	0.16 (-0.22 to 0.50)	-0.15 (-0.49 to 0.23)	-0.17 (-0.51 to 0.21)	-0.02 (-0.38 to 0.35)
Right hemisphere				
MFG	0.33 (-0.04 to 0.62)	-0.18 (-0.51 to 0.20)	-0.01 (-0.37 to 0.36)	-0.17 (-0.50 to 0.21)
IFG	-0.01 (-0.38 to 0.36)	0.12 (-0.26 to 0.47)	-0.05 (-0.41 to 0.32)	-0.12 (-0.47 to 0.26)
STS	0.33 (-0.05 to 0.62)	-0.24 (-0.55 to 0.14)	0.12 (-0.26 to 0.47)	-0.16 (-0.50 to 0.22)
IPL	0.35 (-0.02 to 0.64)	-0.04 (-0.40 to 0.33)	-0.14 (-0.48 to 0.24)	-0.10 (-0.45 to 0.28)
SP group				
Left hemisphere				
MFG	0.24 (-0.15 to 0.56)	0.23 (-0.15 to 0.56)	**0.55 (0.23 to 0.77)***	0.08 (-0.30 to 0.44)
IFG	0.29 (-0.10 to 0.60)	-0.07 (-0.43 to 0.31)	0.37 (-0.01 to 0.65)	-0.24 (-0.56 to 0.15)
STS	0.11 (-0.28 to 0.46)	-0.12 (-0.47 to 0.27)	0.21 (-0.18 to 0.54)	0.08 (-0.30 to 0.44)
IPL	0.13 (-0.26 to 0.48)	0.15 (-0.23 to 0.50)	0.12 (-0.27 to 0.47)	-0.11 (-0.46 to 0.28)
Right hemisphere				
MFG	0.21 (-0.17 to 0.54)	0.27 (-0.11 to 0.59)	0.16 (-0.23 to 0.50)	-0.07 (-0.43 to 0.31)
IFG	0.30 (-0.08 to 0.61)	0.31 (-0.07 to 0.61)	0.13 (-0.26 to 0.48)	0.05 (-0.33 to 0.42)
STS	-0.13 (-0.48 to 0.26)	-0.09 (-0.45 to 0.29)	0.13 (-0.25 to 0.48)	-0.06 (-0.42 to 0.32)
IPL	0.03 (-0.35 to 0.40)	-0.07 (-0.43 to 0.32)	0.00 (-0.37 to 0.38)	-0.14 (-0.49 to 0.25)

R values (and their 95% confidence intervals) are presented. Δ indicates changes of behavioral performance after intervention. * indicates a p-value < 0.05.
